# Genetic dissection of cardiac iron regulation using transcriptome network analysis and systems genetics in BXD mice

**DOI:** 10.1016/j.xhgg.2026.100668

**Published:** 2026-09-01

**Authors:** Akhilesh K. Bajpai, Qingqing Gu, Rakesh Kumar, Xiangtang Li, Athena Starlard-Davenport, Wenjing Zhang, Enkhsaikhan Purevjav, Byron C. Jones, Qingyin Zheng, Dong Wang, Lu Lu

**Affiliations:** 1 Department of Otolaryngology Head and Neck Surgery, Institute of Otology and Neurobiology, Shandong Medical and Pharmaceutical University Hospital, Binzhou, Shandong 256603, China; 2 Department of Genetics, Genomics and Informatics, University of Tennessee Health Science Center, Memphis, TN 38163, USA; 3 Affiliated Hospital of Nantong University, Department of Cardiology, Nantong, Jiangsu 226000, China; 4 Department of Anatomy, Kasturba Medical College, Manipal Academy of Higher Education, Manipal, India; 5 Department of Pathology and Laboratory Medicine, University of Tennessee Health Science Center, Le Bonheur Children’s Hospital, Memphis, TN 38103, USA; 6 Department of Pathology, University of Tennessee Health Science Center, Memphis, TN 38163, USA; 7 Department of Pediatrics, University of Tennessee Health Science Center, Memphis, TN 38163, USA; 8 Shandong Key Lab of Complex Medical Intelligence and Aging, School of Special Education and Rehabilitation, Shandong Medical and Pharmaceutical University, Yantai, Shandong 264003, China; 9 Department of Cardiology, Shandong Medical and Pharmaceutical University Hospital, Binzhou, Shandong 256603, China

**Keywords:** BXD mouse population, systems genetics, cardiac iron homeostasis, QTL mapping, WGCNA, cardiomyopathy

## Abstract

Cardiac iron homeostasis is essential for myocardial energy metabolism and contractile function, yet the genetic and molecular mechanisms governing iron levels within the heart remain poorly understood. We used a systems genetics approach to dissect the transcriptional regulation of cardiac iron homeostasis. Myocardial iron level varies substantially across BXD strains (40–112 μg/g) and is under heritable genetic control (H^2^ = 0.38). Elevated cardiac iron is associated with reduced ventricular mass, increased ventricular ectopy, and prolonged atrioventricular conduction in the BXD population. Weighted gene co-expression network analysis of the BXD heart transcriptome identified a co-expression module that was significantly and negatively correlated with cardiac iron levels in both young and old BXD mice and enriched for pathways related to metabolic regulation, cyclic AMP (cAMP) signaling, circadian entrainment, and cardiovascular physiology. The module showed substantial overlap with a curated cardiac iron gene set, and cross-species enrichment analysis confirmed its conservation in human cardiomyopathy differentially expressed genes (enrichment ratio = 1.49; false discovery rate [FDR] = 0.0342). Quantitative trait locus (QTL) mapping of the first principal component of the overlapping module iron genes (*n* = 38), corroborated by individual gene mapping, identified *trans*-eQTL hotspots on multiple chromosomes, implicating *Fcho2*, *Gcc2*, and *Rmdn1* as candidate upstream regulators operating through sequential steps of intracellular iron trafficking. Together, these findings establish a systems-level map of cardiac iron gene regulation, identify candidate genetic regulators, and provide a molecular framework linking disruption of iron-related transcriptional networks to structural and electrical cardiac dysfunction with implications for iron-related heart diseases.

## Teaser

We identified transcriptional networks and candidate genetic regulators that control cardiac iron homeostasis using systems genetics in BXD mice. These findings provide a systems-level framework linking iron regulation to cardiac structure and electrical function, offering new insights into the genetic basis of iron-related heart disease.

## Introduction

Iron is an essential trace element that plays a central role in a wide range of biological processes, including oxygen transport, mitochondrial respiration, DNA synthesis, and cellular metabolism.^1^^,^^2^ Its redox-active properties enable iron to function as a critical cofactor in heme- and iron-sulfur cluster-containing proteins that drive oxidative phosphorylation and enzymatic reactions.^3^ However, these same properties render iron potentially toxic, as excess labile iron catalyzes the formation of reactive oxygen species (ROSs), leading to oxidative stress, lipid peroxidation, and cellular damage.^4^^,^^5^ Consequently, iron homeostasis must be tightly regulated to balance its essential biological functions against its cytotoxic potential.

Systemic iron homeostasis is maintained through coordinated regulation of iron absorption, recycling, storage, and export, primarily governed by the hepcidin-ferroportin axis.^6^^,^^7^ Hepcidin, a liver-derived peptide hormone, controls plasma iron levels by binding to the iron exporter ferroportin, inducing its internalization and degradation, thereby limiting iron efflux from enterocytes, macrophages, and hepatocytes.^8^ Ferroportin itself is indispensable for systemic iron export, as its loss leads to severe iron retention in absorptive and recycling cells.^9^ At the cellular level, iron uptake is mediated by transferrin receptor 1 (TfR1) and divalent metal transporter 1 (DMT1), while intracellular storage and utilization are regulated by ferritin and mitochondrial pathways.^10^^,^^11^ Iron regulatory proteins (IRP1/2) further fine-tune this system by post-transcriptionally controlling the expression of key iron-metabolism genes in response to intracellular iron levels.^12^ Together, these multi-layered regulatory mechanisms ensure that iron availability remains within an optimal physiological range.

The heart is particularly sensitive to disruptions in iron homeostasis due to its high energetic demands and dependence on mitochondrial oxidative metabolism.^13^ Both iron deficiency and iron overload have been strongly associated with impaired cardiac function. Iron deficiency impairs mitochondrial respiration and reduces ATP production in cardiomyocytes, leading to decreased contractility,^14^ and has been independently associated with worse outcomes in patients with chronic heart failure.^15^^,^^16^ In contrast, iron overload promotes excessive ROS generation, disrupts calcium handling, and induces cardiomyocyte injury, leading to cardiomyopathy and arrhythmias.^17^^,^^18^ In addition, ferroptosis, an iron-dependent form of regulated cell death characterized by lipid peroxidation, has emerged as a key mechanism linking iron dysregulation to cardiac injury in ischemia-reperfusion and heart failure.^19^^,^^20^ These findings highlight the importance of maintaining precise iron balance in cardiac tissue. Beyond systemic regulation, emerging evidence indicates that cardiomyocyte-intrinsic mechanisms play a critical role in maintaining intracellular iron balance. Notably, ferroportin expression within cardiomyocytes is essential for preventing iron accumulation, and its deletion leads to severe cardiac dysfunction independent of systemic iron levels.^21^ Furthermore, intracellular iron compartmentalization—particularly its distribution between cytosolic and mitochondrial pools—has been shown to be a key determinant of cardiac pathology.^22^ These observations suggest that local regulatory networks within the heart are critical for fine-tuning iron homeostasis and may be influenced by genetic variation.

Despite significant advances in understanding iron metabolism, the genetic determinants underlying variability in cardiac iron levels remain poorly defined. In particular, the identification of modifier genes that regulate iron handling within cardiac tissue has been limited. Human genome-wide association studies (GWASs) have identified several loci influencing systemic iron traits, including variants near *TMPRSS6*, *TF*, *TFR2*, and *HFE*, highlighting a polygenic architecture for iron regulation.^23^ However, these studies predominantly capture variation in circulating iron indices and do not resolve tissue-specific regulatory mechanisms, particularly within the heart. Moreover, while epidemiological evidence links iron dysregulation to cardiac disease, establishing causality remains challenging due to confounding factors. Systems genetics approaches provide a powerful framework to address this gap by integrating genetic variation, gene expression, and phenotypic traits.^24^ The BXD recombinant inbred mouse panel represents a well-established genetically diverse population model for dissecting complex traits, enabling the identification of quantitative trait loci (QTLs) and candidate genes that contribute to physiological variation,^25^ including cardiovascular abnormalities and ion homeostasis.^26^^,^^27^^,^^28^

In this study, we applied an integrative systems genetics approach to identify genetic regulators of the cardiac iron gene-network in BXD mice. By combining co-expression network analysis, QTL mapping, transcriptomic correlation analysis, and human causal inference through Mendelian randomization, we aimed to uncover candidate genes and molecular pathways that modulate cardiac iron homeostasis. Our findings provide insights into the genetic architecture of iron regulation in the heart and highlight coordinated networks linking iron uptake, intracellular trafficking, and mitochondrial utilization to cardiac function.

## Material and methods

### Animals

All animal studies were approved by the Institutional Animal Care and Use Committee (IACUC) of the University of Tennessee Health Science Center (UTHSC). BXD mice were maintained in microisolator cages at 25°C under a 12-h light/12-h dark cycle with free access to water and food. For electrocardiogram (ECG) and echocardiographic analyses, we used male and female mice from the parental B6 and D2 strains and 42 BXD RI strains (*n* ≥ 5 mice/strain/sex) at 4–5 months of age. For RNA sequencing (RNA-seq), we used 73 BXD RI strains and the two parental strains. All mice were maintained under standard laboratory conditions, remained sedentary, and were fed a standard chow diet (2018 Teklad Global 18% protein, Teklad Diets, Madison, WI). Cardiac iron phenotypes were obtained from a cohort comprising 41–83 BXD RI strains, depending on the phenotype analyzed. For all integrative analyses, only strains common to the corresponding transcriptomic and phenotypic datasets were included.

### ECG tracing

Following anesthesia with 2% isoflurane, subcutaneous needle electrodes were placed in the left forelimb and hindlimb to obtain consistent single-lead ECG recordings while mice were maintained on a heated pad. Animals were kept under a stable 2% isoflurane supply in 100% oxygen throughout the recording period to minimize variability. ECG signals were acquired for 5 min at a sampling rate of 2000 Hz using a BIOPAC system (Goleta, CA) with AcqKnowledge 3.9.2 software. Recorded data were subsequently exported to LabChart 7 for analysis.

Standard ECG parameters, including P wave, PR interval, QRS complex, and QT interval, were measured from clearly defined waveforms. RR interval scatterplots were generated automatically, and heart rate variability was assessed from the RR intervals over the 5-min recording period. Normal and arrhythmic rhythms were identified based on RR interval variability, as previously described.^29^ Arrhythmic events were independently evaluated and classified by two blinded investigators. The frequency of arrhythmias was expressed per thousand (‰) beats, calculated as the number of abnormal events divided by the total number of heartbeats during the recording period, multiplied by 1,000.

### Blood pressure and echocardiography parameters

Blood pressure was measured using a noninvasive tail-cuff system (CODA, Kent Scientific Corporation), which determines systolic and diastolic pressure based on tail blood volume. Mice were acclimated to the restraint device for 15 min daily over three consecutive days prior to measurements. On the day of recording, animals were allowed an additional 5-min acclimation period before data acquisition. Each session consisted of 15 measurement cycles, of which the first five were considered acclimation cycles and excluded from analysis; only valid cycles were used for final calculations.

Cardiac structure and function were assessed by transthoracic two-dimensional and Doppler echocardiography using a Vevo 2100 imaging system (VisualSonics, Toronto, Canada), as previously described.^28^

### Cardiac iron phenotypes measurement in BXD mice

Briefly, BXD mice were housed under standardized conditions and fed a defined rodent diet (Envigo 7912) containing fixed trace metal concentrations (240 ppm Fe, 93 ppm Cu, and 63 ppm Zn) to minimize environmental variability in metal exposure. Heart tissue was wet-digested in trace-metal-grade nitric acid and analyzed for iron (Fe) content by total reflection X-ray fluorescence (TXRF) spectroscopy using an S2 PICOFOX instrument (Bruker, Berlin). Digested samples were spiked with gallium (Ga) as an internal standard, deposited onto quartz sample carriers, and measured for 500 s. Fe concentrations were determined by internal standard normalization relative to Ga and normalized to tissue wet weight. Iron concentration (μg/g) is reported as mean ± standard error of the mean (SEM).

Phenotypic values were calculated at the strain level and represent mean iron concentrations across individuals within each strain (*n* = 41–83 strains per trait). Four cardiac iron traits were analyzed and are publicly accessible via GeneNetwork under the following identifiers: BXD_21394 (males, 20–650 days), BXD_21397 (females, 20–650 days), BXD_21400 (combined sexes, 20–240 days), and BXD_21518 (combined sexes, 20–650 days). A total of 456 animals were used for profiling cardiac iron levels. The day of birth was defined as day 0. Detailed information for each phenotype, including the number of strains, number of animals, and median age, is provided in Table S1. For simplicity, the BXD_21400 phenotype is referred to as the younger-age trait, whereas BXD_21394, BXD_21397, and BXD_21518 are referred to as older-age traits based on their substantially higher median ages (312–330 days vs. 179 days), rather than their nominal age ranges.

### RNA extraction and tissue collection

The RNA-seq data were generated from a total of 73 BXD strains and both parental strains (C57BL/6J and DBA/2J). Animals were euthanized under saturated isoflurane anesthesia, and whole hearts were rapidly excised. The tissues were immediately frozen and stored at −80°C until further processing. For RNA isolation, approximately 30 mg of left ventricular tissue was homogenized in 700 μL QIAzol Lysis Reagent (Qiagen, Hilden, Germany) using a 5-mm stainless steel bead in a TissueLyser II (Qiagen) at 30 Hz for 2 min, followed by a 5-min incubation at room temperature. Subsequently, 140 μL of chloroform was added, and samples were vigorously shaken for 15 s before centrifugation at 12,000 × *g* for 15 min at 4°C. The aqueous phase (∼280 μL) was transferred to a new tube and mixed with 500 μL of 100% ethanol. The mixture was then loaded onto an RNeasy Mini spin column (Qiagen, Valencia, CA, USA) and purified using Buffer RWT and Buffer RPE according to the manufacturer’s instructions. RNA quality and integrity were assessed using the Agilent 2100 Bioanalyzer (Agilent Technologies, Santa Clara, CA, USA), and only samples with an OD260/280 ratio >1.8 and RNA integrity number (RIN) >8.0 were used for downstream library preparation.

### Library preparation, RNA-seq, and data analysis

For RNA-seq, one microgram of total RNA per sample was used for cDNA library preparation using the NEBNext Ultra RNA Library Prep Kit for Illumina (New England Biolabs, Ipswich, MA, USA) following the manufacturer’s protocol. Briefly, mRNA was enriched using oligo(dT) magnetic beads and subjected to two rounds of purification, followed by fragmentation. First-strand cDNA synthesis was performed using random hexamer primers, and second-strand cDNA synthesis was carried out using a custom buffer containing dNTPs, RNase H, and DNA polymerase I. The resulting double-stranded cDNA underwent end repair, A-tailing, and adapter ligation, followed by size selection and PCR amplification to generate final libraries with insert sizes ranging from 250 to 350 bp. Library concentration was quantified using a Qubit 2.0 fluorometer (Thermo Fisher Scientific, Waltham, MA, USA), and size distribution was assessed using the Agilent 2100 Bioanalyzer. The libraries were sequenced on an Illumina NovaSeq platform using paired-end 150 bp reads (2 × 150), resulting in an average of ∼40 million raw reads per sample.

Raw sequencing reads were aligned to the mouse reference genome (GRCm38) obtained from the Ensembl genome browser (https://www.ensembl.org/) using STAR aligner (v2.5.0a).^30^ Genome indices were generated prior to alignment, and STAR’s Maximal Mappable Prefix algorithm was employed to ensure accurate mapping, particularly across splice junctions. Gene-level read counts were quantified using FeatureCounts (v0.6.1).^31^ Expression levels were normalized as transcripts per million (TPM). For downstream analyses, TPM values were log-transformed as log_2_(TPM + 1) to stabilize variance and improve comparability across samples. The normalized data can be accessed through GeneNetwork (GN1028: NHLBI BXD All Ages Heart RNA-Seq (Nov20) TPM Log2).

### Heritability estimation

We estimated the broad-sense heritability (H^2^) for the iron phenotypes using ANOVA-based variance decomposition. Because each phenotype represented a predefined dataset with a specific age and/or sex composition, age and sex were not included as covariates in the variance decomposition model. We reconstructed within-strain variance as *SE*^2^ × *n* and calculated pooled environmental variance (*Ve*) as the weighted average of within-strain variances. Between-strain mean squares were computed from sample size-weighted deviations from the grand mean, and genetic variance (*Vg*) was estimated as $\left(MS_{\text{between}}-Ve\right)/\overset{ˉ}{n}$, where $\overset{ˉ}{n}$ is the average strain size. Broad-sense heritability was calculated as

$$H^{2}=Vg/\left(Vg+Ve\right)\text{.}$$

Ninety-five percent confidence intervals were obtained using a parametric bootstrap (1,000 iterations). All analyses were performed in R.

### Weighted gene co-expression network analysis

Weighted gene co-expression network analysis (WGCNA) was performed on the top 10,000 variance-based genes from the GN1028 Heart BXD dataset (NHLBI BXD All Ages Heart RNA-Seq Nov20 TPM Log2) using the WGCNA R package.^32^ Hierarchical clustering of samples was performed using the *hclust* function (average linkage, Euclidean distance) to identify outlier strains, which were removed prior to network construction to improve robustness (Figure S1). Phenotypic trait data were aligned to the expression matrix using common strain identifiers, and only overlapping samples were retained for downstream analysis. A signed co-expression network was constructed by calculating an adjacency matrix using a soft-thresholding power (β) of 12, selected using *pickSoftThreshold* based on the scale-free topology criterion and consideration of mean connectivity. The adjacency matrix was transformed into a topological overlap matrix (TOM), and the corresponding dissimilarity (1 − TOM) was used for hierarchical clustering of genes. Gene modules were identified using the dynamic tree cut algorithm (*cutreeDynamic*) with parameters *deepSplit*= 3 and *minimum module size* = 30. Highly similar modules were merged using *mergeCloseModules* with a cut height of 0.10. Module-trait relationships were evaluated by correlating module eigengenes (MEs) with four iron-related phenotypes (BXD_21394, BXD_21397, BXD_21400, and BXD_21518) using Pearson correlation, with statistical significance assessed using Student’s asymptotic *p* values and adjusted for multiple testing using the false discovery rate (FDR). Gene-level metrics were calculated, including module membership (MM), defined as the correlation between gene expression and MEs, and gene significance (GS), defined as the correlation between gene expression and phenotypic traits. Intramodular connectivity was computed to further characterize network topology.

### Curation of iron homeostasis gene set

A comprehensive list of iron homeostasis-related genes was assembled by integrating gene sets retrieved from four independent databases: Gene Ontology (GO: http://geneontology.org/),^33^ GeneCards (https://www.genecards.org/),^34^ the Kyoto Encyclopedia of Genes and Genomes (KEGG: https://www.genome.jp/kegg/),^35^ and FerrDB (https://www.zhounan.org/ferrdb/).^36^

GO terms directly relevant to iron biology, including iron uptake, transport, homeostasis and metabolism were queried to retrieve mouse and human gene annotations. From GeneCards, genes were retrieved using the search query terms, such as “iron homeostasis,” “iron metabolism,” “iron level,” “ferroptosis,” “iron uptake,” and “iron transport,” and those with a relevance score greater than 5 were retained. From KEGG, genes associated with iron-relevant pathways were manually curated, including pathways related to ferroptosis, mineral absorption, porphyrin metabolism, mitochondrial iron regulation, lysosomal function, HIF-1 signaling, oxidative stress, and endocytosis. From FerrDB, a database dedicated to ferroptosis regulators, genes classified as ferroptosis drivers, markers, suppressors, and unclassified ferroptosis-associated genes were retrieved and combined into a unified FerrDB gene list. The human genes were subsequently mapped to their mouse orthologs.

Gene lists from all four databases were merged and deduplicated to generate a comprehensive iron homeostasis gene set comprising 1,868 unique mouse genes. To ensure biological relevance in the cardiac context, this combined list was cross-referenced against the BXD heart RNA-seq dataset (GN1028: NHLBI BXD All Ages Heart RNA-Seq Nov20 TPM Log2) and genes with no expression information were excluded, yielding a final filtered iron gene set of 1,279 genes.

### Functional enrichment analysis

Functional enrichment analysis of the genes was performed using WebGestalt (WEB-based Gene SeT AnaLysis Toolkit: https://www.webgestalt.org/)^37^ and Enrichr (https://maayanlab.cloud/Enrichr).^38^ KEGG pathway analysis, Mouse Phenotype Ontology (MPO) annotation, and GO biological process enrichment were conducted using WebGestalt, applying the over-representation analysis (ORA) method with the mouse protein-coding gene set as the reference. Disease association and cell-type enrichment analyses were performed using Enrichr. Disease associations were assessed using the DisGeNET gene-disease association database (https://disgenet.com/),^39^ which integrates curated and text-mined associations between genes and human diseases. Cell-type enrichment was performed using the Tabula Muris gene set library,^40^ a comprehensive single-cell RNA-seq atlas of mouse tissues, to identify cell populations whose transcriptional signatures are overrepresented among the module genes. Terms with an FDR-adjusted *p* < 0.1 were considered statistically significant.

### QTL mapping

To identify genetic loci regulating iron-related transcriptional variation in the heart, a gene-set-based QTL mapping approach was employed using expression data from the BXD mouse reference population. Iron-related genes identified within the yellow WGCNA module were used for QTL mapping. Expression values for the iron-related genes were extracted from the BXD heart RNA-seq dataset (GN1028: NHLBI BXD All Ages Heart RNA-Seq Nov20 TPM Log2) available through GeneNetwork. The first principal component (PC1) was calculated from the expression matrix of these genes across all BXD strains using principal component analysis with mean centering and unit variance scaling, capturing the major axis of coordinated transcriptional variation within the iron gene set. The resulting PC1 scores were uploaded to GeneNetwork as a quantitative trait for genome-wide QTL mapping. Genome-wide interval mapping was performed using the GEMMA algorithm^41^ with leave-one-chromosome-out (LOCO) kinship matrix correction to account for population structure within the BXD panel, using the WGS-based genotype file (Mar2022). A threshold of –log_10_*P* > 3.5 was applied as the genome-wide significance cutoff, consistent with standard criteria for QTL mapping in the BXD panel.

To validate and interpret gene-set-level findings, individual gene QTL mapping was performed for each of the 38 iron-related yellow-module genes using the same dataset, genotype file, and GEMMA mapping framework within GeneNetwork. Peak QTL positions, –log_10_*P*, chromosomal locations, and effect sizes were recorded for each gene. Genes sharing peak QTL locations within a defined genomic interval were grouped to identify *trans*-expression QTL (eQTL) hotspots—genomic loci regulating the expression of multiple iron-related genes simultaneously—suggestive of master regulatory loci controlling cardiac iron gene expression. *cis*-eQTLs were defined as peak QTL positions falling within 5 Mb of the gene’s physical genomic location, while peaks outside this window were classified as *trans*-eQTLs.

### Differential gene expression analysis using human cardiomyopathy samples

Differential expression analysis was performed on a human cardiomyopathy RNA-seq dataset (GSE55296)^42^ comprising 12 dilated cardiomyopathy (DCM) and 10 healthy control samples. Raw transcript counts were processed using the DESeq2 package (v1.50.2)^43^ in R. Genes with fewer than 10 total reads summed across all samples were excluded prior to analysis to reduce low-count noise and improve statistical robustness. The experimental design accounted for both biological condition and batch effects using the model *design = ∼ batch + condition*, enabling adjustment for technical variation while testing for differential expression between DCM and control groups. Library depth normalization was performed by estimating size factors, and gene-wise dispersion was modeled under a negative binomial framework. Differential expression statistics, including log2 fold changes and Wald test *p* values, were extracted using the DESeq2 *results()* function. *p* values were adjusted for multiple comparisons using the Benjamini-Hochberg procedure to control the FDR, and genes with an FDR-adjusted *p* < 0.1 and an absolute fold change ≥1.5 were considered differentially expressed.

### Candidate gene selection

To identify candidate master regulators of cardiac iron homeostasis, we defined 1.5-LOD support intervals for each QTL region and extracted all protein-coding genes within these intervals. We then prioritized candidates by integrating multiple lines of functional and genetic evidence: (1) functional relevance based on curated iron-metabolism gene sets, (2) Pearson correlation between gene expression and iron-related phenotypes in the BXD heart dataset, (3) the presence of significant *cis*-eQTLs in cardiac tissue, and (4) the presence of non-synonymous coding SNPs between the B6 and D2 parental strains. Genes supported by any of these criteria were retained for further analysis and subsequently evaluated using two-sample Mendelian randomization (MR) with human heart eQTL data from the GTEx project (v11). Final candidate genes were selected based on the convergence of evidence, including strong mouse genetic support (high –log_10_*P* and phenotype correlations), functional annotation, and significant causal effects in human MR analyses (uncorrected *p* < 0.05).

### MR

We used a prioritized list of candidate genes derived from mapped QTL intervals for MR analysis. Gene symbols were standardized and converted to human gene identifiers using the org.Hs.eg.db annotation database in R to ensure compatibility with GTEx datasets. Significant *cis*-eQTL summary statistics were obtained from the GTEx v11 database (https://gtexportal.org/)^44^ for three cardiovascular-relevant tissues: left ventricle, atrial appendage, and coronary artery. Variant-gene pairs corresponding to the prioritized genes were extracted from GTEx parquet datasets, and variant identifiers were harmonized and mapped to dbSNP reference SNP IDs (rsIDs) using the GTEx whole-genome sequencing lookup table.

For MR analysis, exposure datasets were formatted to include SNP identifiers, effect and non-effect alleles, effect sizes (β), standard errors, allele frequencies, and *p* values. Independent genetic instruments were selected using a significance threshold (*p* < 1 × 10^−5^), followed by linkage disequilibrium (LD) clumping implemented in the TwoSampleMR package^45^ to retain independent variants. Instrument strength was evaluated using the F-statistic (F = β^2^/SE^2^), and all retained instruments had F-statistics >10 (range: 13–1,110 across the three tissue-specific eQTL datasets), indicating adequate instrument strength and a low likelihood of weak instrument bias. Outcome summary statistics were obtained from the Integrative Epidemiology Unit (IEU) OpenGWAS database^45^ and included cardiovascular and iron-related traits, such as heart failure (ebi-a-GCST009541), cardiomyopathy (finn-b-I9_CARDIOMYOPATHY), atrial fibrillation (ebi-a-GCST006414), transferrin levels (ieu-a-1052), serum iron (ieu-a-1049), transferrin saturation (ieu-a-1051), and left ventricular ejection fraction (ebi-a-GCST005913). Exposure and outcome datasets were harmonized to align effect alleles and ensure consistent directionality prior to analysis.

Causal effects were estimated using two-sample MR implemented in TwoSampleMR. For genes with a single genetic instrument, the Wald ratio method was applied, whereas, for genes with multiple independent instruments, the inverse variance weighted (IVW) method was used as the primary analysis. Sensitivity analyses, including heterogeneity testing and MR-Egger intercept analysis, were performed where sufficient independent genetic instruments were available. These analyses were not applicable to single-instrument associations, which were therefore interpreted with caution. Primary MR results were presented using nominal significance (*p* < 0.05). To assess the robustness of these findings, *p* values from all MR analyses within each tissue were subsequently adjusted using the Benjamini-Hochberg method (FDR-adjusted *p* < 0.10). Results were reported as effect estimates and corresponding odds ratios where appropriate. All analyses were conducted in R using standard pipelines.

### Candidate gene–correlated transcripts and network analysis

To characterize the biological context of prioritized candidate genes, gene-gene correlation analysis was performed using the BXD heart transcriptomic dataset. Top 2,000 genes showing the strongest correlation with gene expression of the candidate genes across strains were selected. These gene sets were subjected to analysis using Metascape (https://metascape.org/)^46^ with default parameters, focusing on GO (biological process) annotations. Enriched terms were filtered based on the default significance threshold (*p* < 0.01) and clustered using a similarity-based approach (kappa statistics) to group related biological processes. Network visualizations were generated in Cytoscape (http://cytoscape.org/),^47^ where nodes represent enriched terms and edges indicate shared gene overlap, allowing identification of functionally coherent modules associated with each candidate gene.

## Results

### Heart iron levels are associated with cardiac remodeling and electrical activity in BXD mice

**Figure 1:**
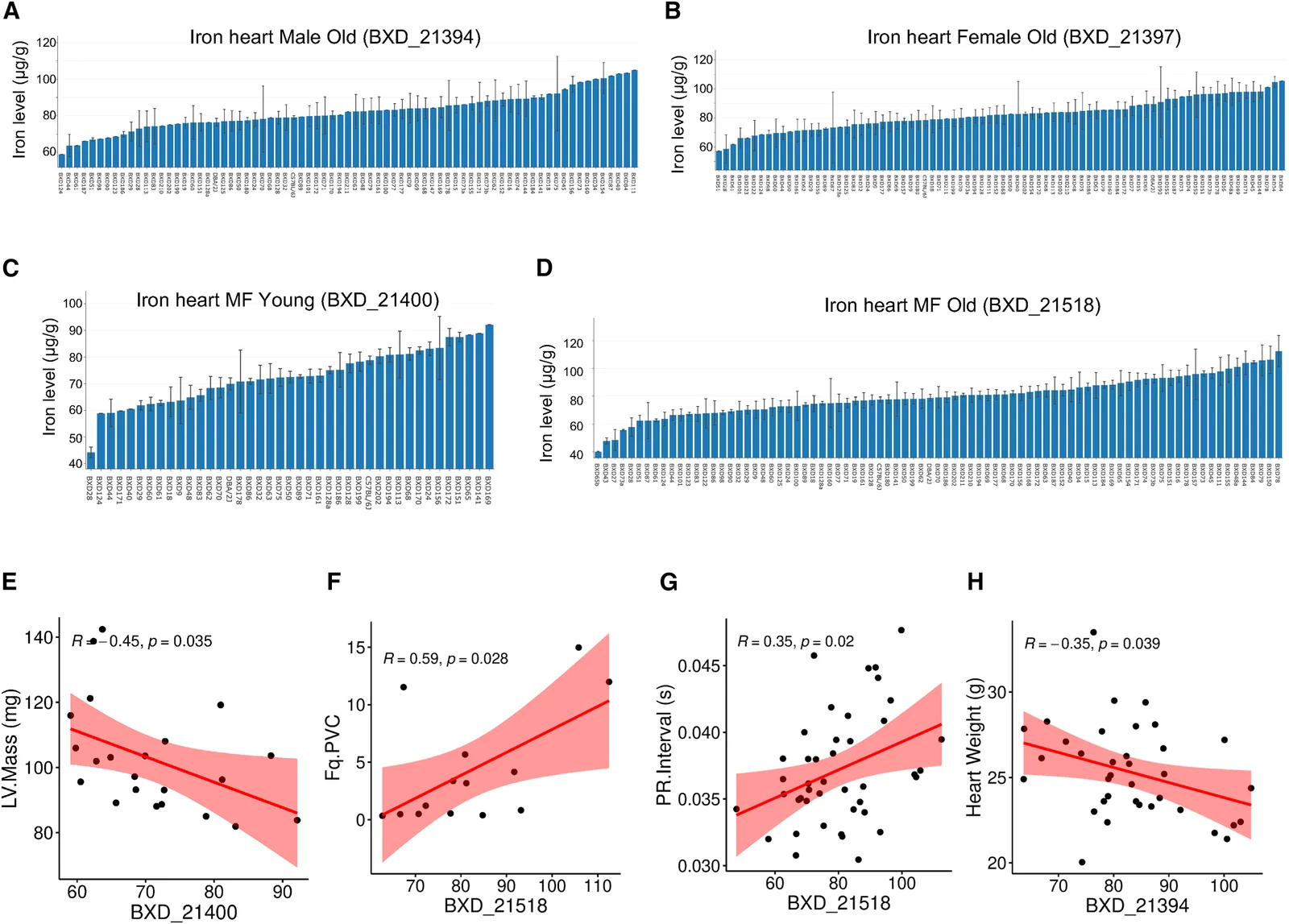
Cardiac iron levels and their association with cardiac phenotypes across BXD strains (A–D) Distribution of cardiac iron levels across BXD strains in four independent cohorts: male old (BXD_21394), female old (BXD_21397), mixed-sex young (BXD_21400), and mixed-sex old (BXD_21518) mice. The terms young and old refer to the overall age distribution of animals contributing to each phenotype rather than strict age cutoffs. The median ages of animals contributing to these phenotypes were 312, 330, 179, and 322 days for male old, female old, mixed-sex (MF), young, and MF old, respectively (Table S1). Bars represent strain means, and error bars indicate variability within strains. (E–H) Correlation of cardiac iron levels with selected echocardiographic and electrocardiographic traits, (E) left ventricular (LV) mass, (F) premature ventricular contraction (PVC) frequency, (G) PR interval duration, and (H) heart weight. The *x* axis indicates iron levels (μg/g) and the *y* axis indicates the cardiac parameters. Red lines indicate linear regression fits with shaded 95% confidence intervals.

Strain-level variation in cardiac iron content was observed across all BXD cohorts, spanning a broad quantitative range in both sexes and age groups (Figures 1A–1D). In male old mice (trait ID: BXD_21394), cardiac iron levels ranged approximately from 59 μg/g in BXD124 to 105 μg/g in BXD111, whereas female old mice (trait ID: BXD_21397) showed a similar but slightly narrower distribution of 57 μg/g in BXD51 to 106 μg/g in BXD84. In mixed-sex young mice (trait ID: BXD_21400), values ranged from 44 μg/g in BXD28 to 92 μg/g in BXD169, while mixed-sex old mice (trait ID: BXD_21518) exhibited a wider spread from 40 μg/g in BXD65b to 112 μg/g in BXD78. This pattern indicates that cardiac iron is a quantitative trait influenced by genetic background. We observed moderate heritability (H^2^) for cardiac iron levels with approximately 40% of the phenotypic variance attributed to genetic differences among the strains.

To assess the relationship between myocardial iron burden and cardiovascular function, we correlated cardiac iron levels measured in male, female, and mixed-sex BXD cohorts with echocardiographic, hemodynamic, and electrocardiographic phenotypes.^27^^,^^28^ Mixed-sex cardiac iron yielded the most consistent associations. In the younger cohort (20–240 days, trait ID: BXD_21400), cardiac iron was significantly negatively correlated with left ventricular (LV) mass (r = −0.45, *p* = 0.035) (Figure 1E), with directionally consistent trends for right ventricular internal diameter (r = −0.41, *p* = 0.085), interventricular septum (IVS), diastole (r = −0.38, *p* = 0.079), IVS-systole (r = −0.36, *p* = 0.09), and cardiac output (r = −0.31), collectively suggesting a dilated or atrophic rather than hypertrophic myocardial response to iron accumulation. Concurrent positive trends for systolic and diastolic blood pressure (r = +0.45 and +0.43, respectively) further support a hemodynamic profile suggestive of iron-overload cardiomyopathy (Data S1). In the older mixed-sex cohort (20–650 days, trait ID: BXD_21518), associations shifted to the electrocardiographic domain, with cardiac iron significantly correlated with premature ventricular contraction frequency (Fq. PVC; r = +0.59, *p* = 0.028) and PR interval duration (r = +0.35, *p* = 0.020), consistent with iron-mediated disruption of ion channel function and atrioventricular conduction (Figures 1F and 1G). Male cardiac iron (20–650 days, trait ID: BXD_21394) showed a significant inverse correlation with heart weight (r = −0.35, *p* = 0.039) and a trend toward increased premature atrial contractions (r = +0.33, *p* = 0.073), extending the iron–arrhythmia association across sexes (Figure 1H and Data S1).

Overall, elevated cardiac iron was associated with two convergent pathological signatures across BXD strains: structural remodeling toward reduced ventricular mass and wall dimensions, and electrophysiological instability including ectopy and conduction delay.

### WGCNA identifies a co-expression module significantly correlated with cardiac iron levels in BXD mice

**Figure 2:**
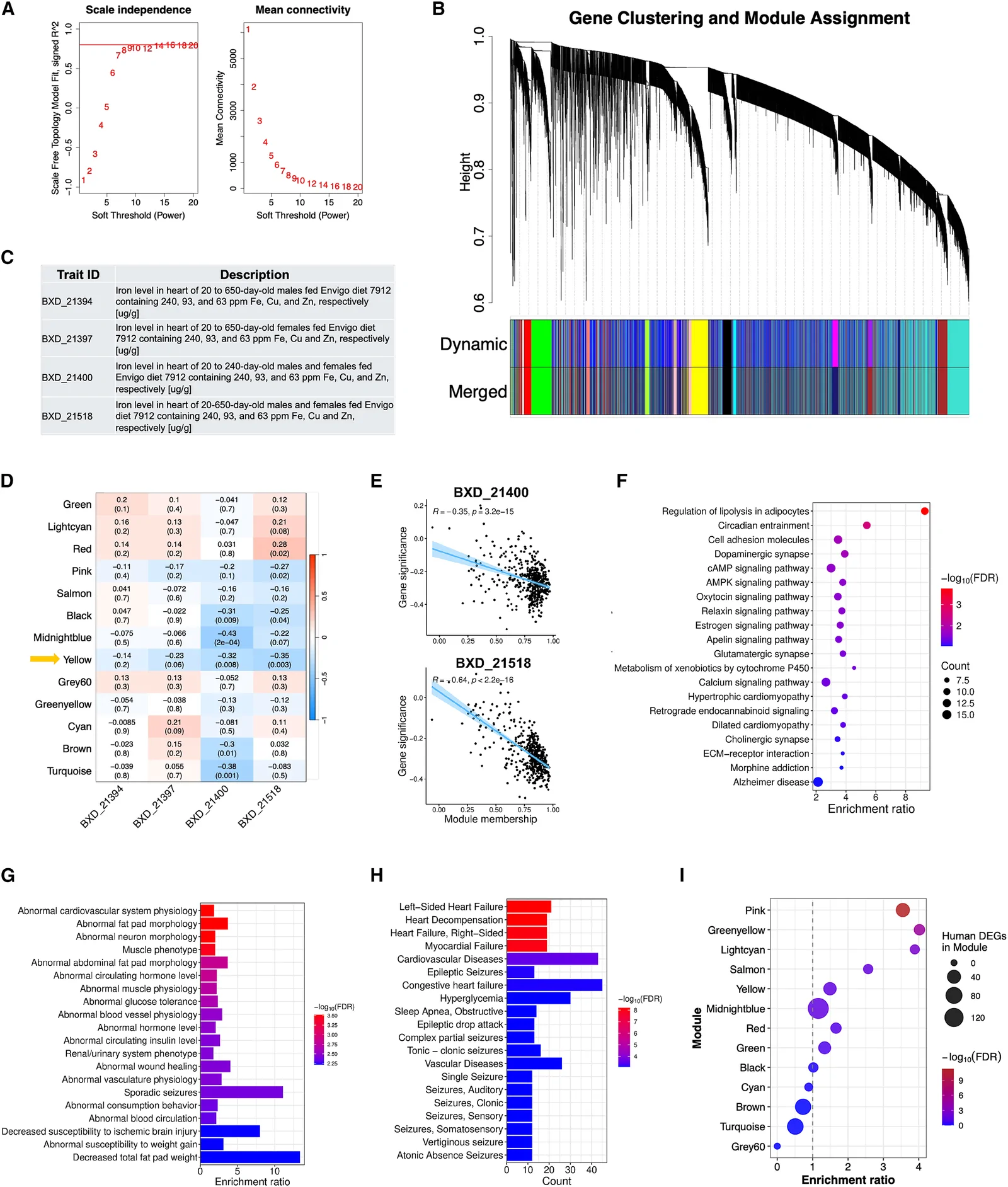
WGCNA and module selection (A) Soft-thresholding power selection for WGCNA network construction. Left: scale-free topology model fit (R^2^, *y* axis) plotted against soft threshold power (*x* axis); the red dashed line indicates the 0.8 threshold, with power = 12 selected as the point at which scale-free topology is achieved. Right: mean network connectivity as a function of soft threshold power. (B) Hierarchical clustering dendrogram of 10,000 highly variable genes based on topological overlap dissimilarity, with dynamic (before merging) and merged module color assignments. Each color band represents a distinct co-expression module identified after merging modules with high similarity (90%). (C) Description of the four BXD phenotypic traits related to heart iron levels used for module-trait correlation analysis. (D) Module-trait correlation heatmap. Each cell displays the Pearson correlation coefficient between a module’s eigengene and each of the four iron-related traits, with the corresponding *r* and *p* indicated in parentheses. Color scale ranges from blue (negative correlation) to red (positive correlation). (E) Module membership (MM) vs. gene significance (GS) scatterplots for the yellow module with respect to BXD_21400 and BXD_21518 traits. Each dot represents a gene with correlation *r* and *p* indicated at the top of each plot. (F) KEGG pathway enrichment dot plot for genes in the yellow module. Dot size reflects the number of genes enriched in each pathway, and dot color indicates statistical significance as −log_10_(FDR). (G) Mouse Genome Informatics (MGI) phenotype enrichment analysis of yellow-module genes. Bar length represents the enrichment ratio and bar color denotes −log_10_(FDR). (H) Disease enrichment analysis of yellow-module genes based on the DisGeNET database. Bar length represents gene counts and bar color indicates −log_10_(FDR). (I) Enrichment of human cardiomyopathy vs. control DEGs across co-expression modules generated from BXD heart transcriptome data. The *x* axis indicates enrichment ratio (dotted line shows enrichment ratio of 1) and *y* axis indicates module names.

To identify gene clusters associated with cardiac iron homeostasis, we performed WGCNA on heart transcriptome data from BXD recombinant inbred strains. A soft-thresholding power of 12 was selected based on the scale-free topology criterion (R^2^ > 0.80) while maintaining adequate mean network connectivity (Figure 2A). Hierarchical clustering of genes based on topological overlap dissimilarity, followed by dynamic tree cutting and module merging, identified 13 distinct co-expression modules, each assigned a unique color (Figure 2B and Data S2).

To relate these modules to physiologically relevant traits, MEs were correlated with four BXD cardiac iron phenotypes (BXD_21394, BXD_21397, BXD_21400, and BXD_21518), representing heart iron levels (μg/g) measured across different age and sex groups under a defined diet (Figure 2C; Table S1). Module-trait correlation analysis revealed that the yellow module (*n* = 472 genes) exhibited a consistent negative correlation with all four cardiac iron traits, with statistically significant associations observed for BXD_21400 (r = −0.32, *p* = 0.008) and BXD_21518 (r = −0.35, *p* = 0.003), identifying it as the primary module of interest (Figure 2D and Data S2). Furthermore, this was the only module that was significantly associated with both of the above cardiac iron traits with an FDR threshold <0.1.

To further validate this association, we assessed the relationship between MM and GS within the yellow module. Both BXD_21400 and BXD_21518 showed significant negative MM-GS correlations (R = −0.35, *p* = 3.2 × 10^−15^ and R = −0.64, *p* < 2.2 × 10^−16^, respectively; Figure 2E), indicating that highly connected hub genes within the module are also the most strongly associated with cardiac iron levels. Notably, the negative direction of these associations suggests that increased expression of yellow-module genes is linked to lower cardiac iron levels, consistent with a potential role in limiting iron accumulation or promoting iron utilization.

### Functional annotation of the co-expression module reveals metabolic, cardiovascular, and neurological enrichment

To characterize the biological functions represented by the yellow-module genes, we performed KEGG pathway, Mouse Genome Informatics (MGI) phenotype, and human disease enrichment analyses (Data S3). KEGG pathway analysis identified significant enrichment in metabolic and signaling pathways, including regulation of lipolysis in adipocytes (gene count = 11, FDR = 1.77E−04), circadian entrainment (gene count = 11, FDR = 0.003), dopaminergic (gene count = 11, FDR = 0.015) and glutamatergic synapse (gene count = 9, FDR = 0.03) signaling, and cyclic AMP (cAMP) signaling (gene count = 14, FDR = 0.022) pathways (Figure 2F), indicating that the yellow module captures broad metabolic and neuroendocrine regulatory processes.

Phenotype enrichment analysis further demonstrated that yellow-module genes are overrepresented among genes associated with abnormal cardiovascular physiology (gene count = 74, FDR = 3.04E−04), altered fat pad and muscle morphology (gene count = 22, FDR = 3.04E−04), impaired glucose tolerance (gene count = 34, FDR = 4.04E−06), abnormal blood circulation (gene count = 36, FDR = 1.58E−05), and reduced susceptibility to ischemic brain injury (gene count = 7, FDR = 2.29E−05) (Figure 2G). Consistent with these findings, human disease enrichment analysis revealed significant associations with cardiovascular conditions, including heart failure, heart decompensation, myocardial failure, and congestive heart failure, as well as metabolic phenotypes such as hyperglycemia and sensory-neural disorders (Figure 2H). Furthermore, cell-type enrichment analysis of the yellow-module genes revealed “endocardial cell heart” as the most significant annotation with 31 genes (FDR = 1.28E−26) (Data S3.

Importantly, these enriched pathways and processes are closely interconnected with iron biology. Iron serves as a critical cofactor in mitochondrial respiration and energy metabolism, and the enrichment of pathways such as cAMP signaling and circadian regulation suggests that the yellow module may coordinate iron-dependent metabolic processes. The strong association with cardiovascular phenotypes and diseases is consistent with the established role of iron in cardiac energetics, mitochondrial function, and oxidative stress. Additionally, enrichment of neuronal signaling pathways likely reflects shared iron-dependent mechanisms underlying mitochondrial activity and redox regulation across tissues.

Together with the enrichment of iron-related genes within this module, these findings support the yellow module as a biologically coherent transcriptional network linking iron homeostasis with metabolic regulation, redox balance, and cardiovascular function.

### Cross-species enrichment of the yellow module with human cardiomyopathy DEGs

To evaluate the translational relevance of mouse co-expression modules, we performed enrichment analysis of differentially expressed genes (DEGs) from a human dilated cardiomyopathy (DCM) dataset across WGCNA modules. Several modules showed significant enrichment; notably, the pink, green-yellow, and light-cyan modules. Importantly, the yellow module, comprising 472 genes, also demonstrated significant enrichment for human DCM-associated DEGs (33 genes; enrichment ratio = 1.49; FDR = 0.0342), indicating a statistically robust, albeit moderate, overlap with human cardiomyopathy-associated transcriptional signatures (Figure 2I). This suggests that the yellow module captures a biologically relevant subset of genes associated with human cardiac disease, in addition to its significant association with cardiac iron levels in BXD mice. Together, these results highlight the yellow module as a biologically meaningful network linking mouse cardiac iron-associated co-expression patterns with broader human cardiomyopathy-related transcriptional alterations.

### Overlap of iron-related genes and functional enrichment link the co-expression module to iron biology

**Figure 3:**
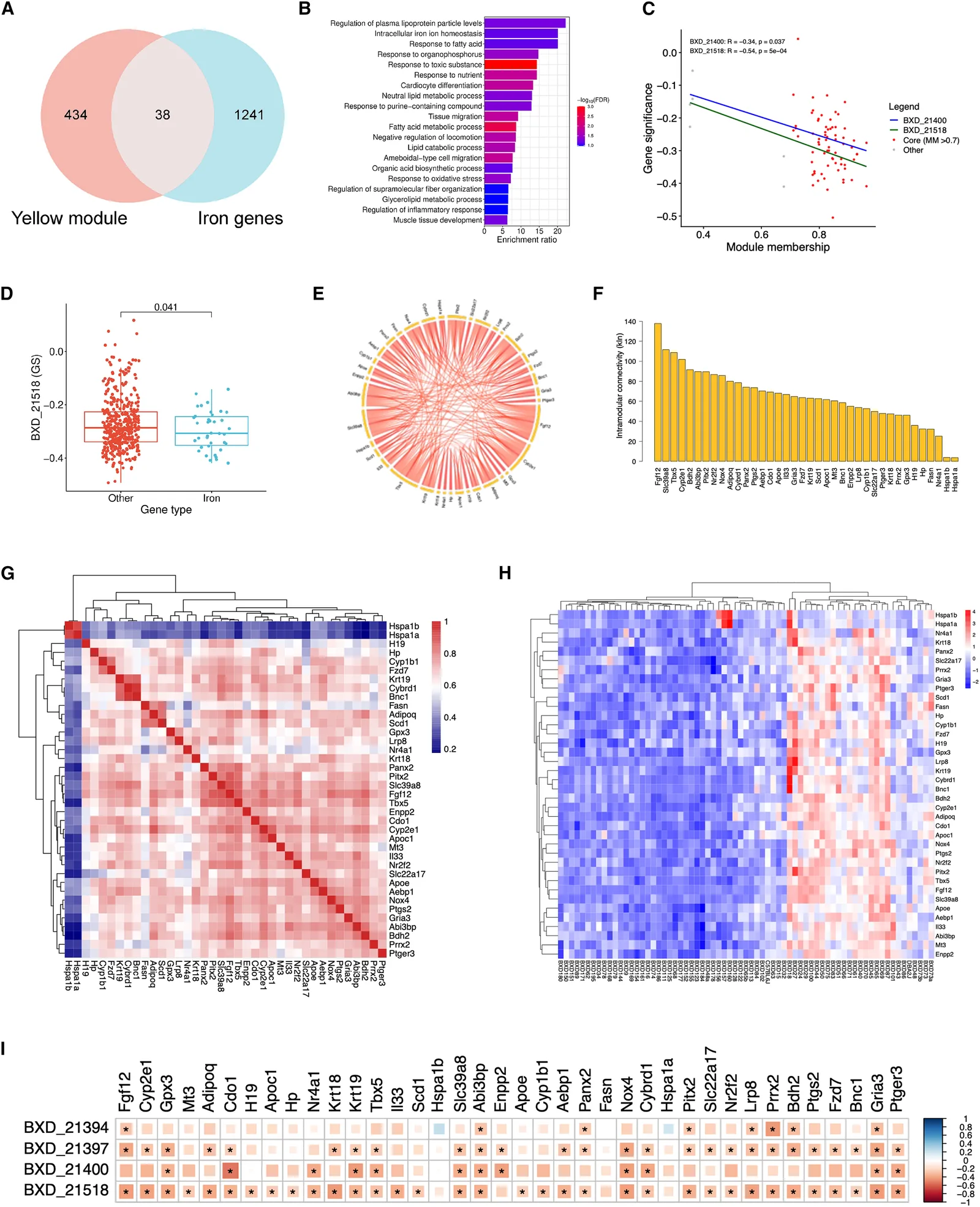
Integrated characterization of iron-related genes within the yellow module (A) Venn diagram showing the overlap between yellow co-expression-module genes (472) and a curated set of iron-related genes (1,279), with 38 genes shared between the two sets. (B) Gene Ontology (GO) biological process enrichment analysis of the 38 overlapping genes. Bar length represents the enrichment ratio, and bar color indicates statistical significance (−log_10_(FDR)), with darker red denoting greater significance. (C) Scatterplot depicting the relationship between module membership (MM) and gene significance (GS) for two BXD traits—BXD_21400 (blue) and BXD_21518 (green)—for the overlapping genes. Core hub iron genes (MM > 0.7) are highlighted in red. (D) Boxplot comparing GS scores for BXD_21518 between non-iron (“other”) and iron-related (“iron”) genes within the yellow module. Iron genes show significantly higher (less negative) GS values compared to other module genes (*p* = 0.041, Student’s *t* test). (E) Circos plot illustrating co-expression connectivity (Pearson correlation analysis) among the iron genes within the yellow module. Each node on the outer ring represents a gene, and arcs represent co-expression edges, with arc density reflecting the degree of inter-gene connectivity. A stringency threshold of |r| > 0.80 was applied. (F) Bar chart ranking yellow-module iron genes by intramodular connectivity (kIn). (G) Heatmap of pairwise Pearson correlation coefficients among the iron genes (within yellow module) across BXD strains. Hierarchical clustering reveals distinct co-expression clusters, with red indicating strong positive correlation and blue indicating negative correlation. (H) Heatmap of normalized expression levels (*Z* scores) for the same 38 overlapping iron genes across individual BXD strains (columns). Red indicates relatively high expression and blue indicates low expression, revealing strain-specific expression patterns among the genes. (I) Pearson correlation coefficients between 38 iron-related genes (columns) and four cardiac iron phenotypes (rows: BXD_21394, BXD_21397, BXD_21400, and BXD_21518). Color scale represents correlation direction and magnitude (blue, positive; red, negative). ∗*p* < 0.05.

To investigate the relationship between the WGCNA-derived yellow co-expression module and iron biology, we compared module genes with a curated set of iron-related genes. Of the 472 genes in the yellow module, 38 overlapped with the iron gene set (Figure 3A).

GO enrichment analysis of the 38 overlapping genes revealed significant enrichment for processes directly related to iron biology, most notably intracellular iron ion homeostasis, alongside lipid metabolic pathways including fatty acid metabolism, lipid catabolism, and glycerolipid metabolism (Figure 3B). Additional enrichment in stress-response and inflammatory pathways—including response to toxic substance, response to oxidative stress, and regulation of inflammatory response—suggests that the functional intersection between the yellow module and iron biology is mediated through shared metabolic and redox-regulatory mechanisms, consistent with the well-established role of iron in mitochondrial function and ROS generation.

To determine whether the overlapping genes reflect biological relevance to cardiac iron levels, we examined the relationship between MM and GS for two iron-related BXD traits. Both BXD_21400 and BXD_21518 showed significant negative correlations between MM and GS (R = −0.34, *p* = 0.037 and R = −0.54, *p* = 5 × 10^−4^, respectively; Figure 3C), indicating that iron genes more central to the yellow module tend to be more strongly negatively associated with cardiac iron phenotypes. Core iron genes (MM > 0.7) were concentrated in the region of highest module membership, further supporting their functional relevance to cardiac iron regulation. The negative direction of these correlations suggests that increased expression of these iron genes is associated with lower cardiac iron levels, pointing to a potential role in regulating iron utilization or limiting iron accumulation in cardiac tissue.

Consistent with this, iron-related genes within the yellow module exhibited significantly higher—that is, less negative—GS values compared to other (non-iron) yellow-module genes (*p* = 0.041; Figure 3D) despite representing a relatively small subset of the module. This finding indicates that iron genes contribute disproportionately to the observed module-trait relationship and likely represent functionally important nodes within the co-expression network.

### Module iron-related genes show high connectivity and strain-specific expression patterns

Network analysis of the yellow-module iron genes revealed a high number of connections among them. Ranking genes by intramodular connectivity identified *Fgf12*, *Slc39a8*, and *Cyp2e1* as the three most highly connected hub genes, followed by *Apoe*, *Ptgs2*, *Nox4*, *Cybrd1*, and *Aebp1*, among others (Figures 3E and 3F).

Pairwise co-expression analysis among the iron genes revealed functional modularity within the broader network, with tightly co-regulated clusters (Figure 3G). For example, one subcluster (*Panx2*, *Pitx2*, *Slc39a8*, *Fgf12*, and *Tbx5*) was enriched for genes involved in cardiac development, transcriptional regulation, and ion transport, suggesting a regulatory layer linking metal homeostasis to cardiac function. A second cluster (*Krt19*, *Cybrd1*, and *Bnc1*) included *Cybrd1*, a key ferric reductase, providing direct evidence of iron-handling mechanisms within the module. A third cluster (*Fasn*, *Adipoq*, and *Scd1*) represented lipid metabolic processes, which are closely connected to iron-dependent mitochondrial function and oxidative stress. Together, these subclusters indicate that the yellow module integrates regulatory, iron-handling, and metabolic pathways, forming a coordinated network underlying cardiac iron homeostasis.

Finally, BXD strains revealed substantial inter-strain variability based on the expression of the overlapping module iron genes, with a subset of strains showing markedly elevated expression of specific gene clusters relative to the majority of the panel (Figure 3H). This heterogeneity likely reflects underlying genetic diversity within the BXD population and may be driven by *cis*- or *trans*-acting regulatory variants that modulate transcriptional activity at these loci in the context of cardiac iron homeostasis.

### Module iron-related genes show significant correlations with BXD cardiac iron levels

Pearson correlation analysis between the heart expression of 38 iron-related yellow-module genes and four cardiac iron phenotype traits revealed a pervasive pattern of negative correlations (Figure 3I), indicating that higher expression of iron-related genes is broadly associated with lower cardiac iron levels across BXD strains.

The number of nominally significant gene-trait correlations (*p* < 0.05) varied markedly across traits, with BXD_21518 showing the most widespread associations (34 of 38 genes), followed by BXD_21397 (26 genes), BXD_21400 (12 genes), and BXD_21394 (eight genes), suggesting that the transcriptional iron response is more strongly coupled to cardiac iron levels in female-inclusive and broader age-spanning cohorts (Figure 3I). After Benjamini-Hochberg correction, all gene-trait associations for BXD_21518 and BXD_21397 remained significant at FDR < 0.1, whereas only one association each was retained for BXD_21394 and BXD_21400 (Data S4).

The strongest individual correlation was observed between *Cdo1* and BXD_21400 (r = −0.53, *p* = 0.002). Other consistently strong associations included *Prrx2* (r = −0.43, BXD_21394), *Nox4* (r = −0.40 to −0.42, three traits), *Fgf12* (r = −0.39 to −0.42, three traits), *Gria3* (r = −0.39 to −0.43, four traits), and *Krt18* (r = −0.43, BXD_21518). Among core iron homeostasis genes, *Cybrd1*, *Slc39a8*, and *Bdh2* showed consistent significant negative correlations across multiple traits, reinforcing their functional relevance to cardiac iron regulation. Notably, *Hspa1b* and *Hspa1a* showed weak positive correlations with the male-specific trait BXD_21394 (r = +0.23 and r = +0.19, respectively), diverging from the otherwise uniform negative pattern (Figure 3I and Data S4) and suggesting a possible iron-induced proteotoxic stress response specific to the male cardiac context.

### QTL mapping identifies three genomic hotspots regulating cardiac iron gene expression

**Figure 4:**
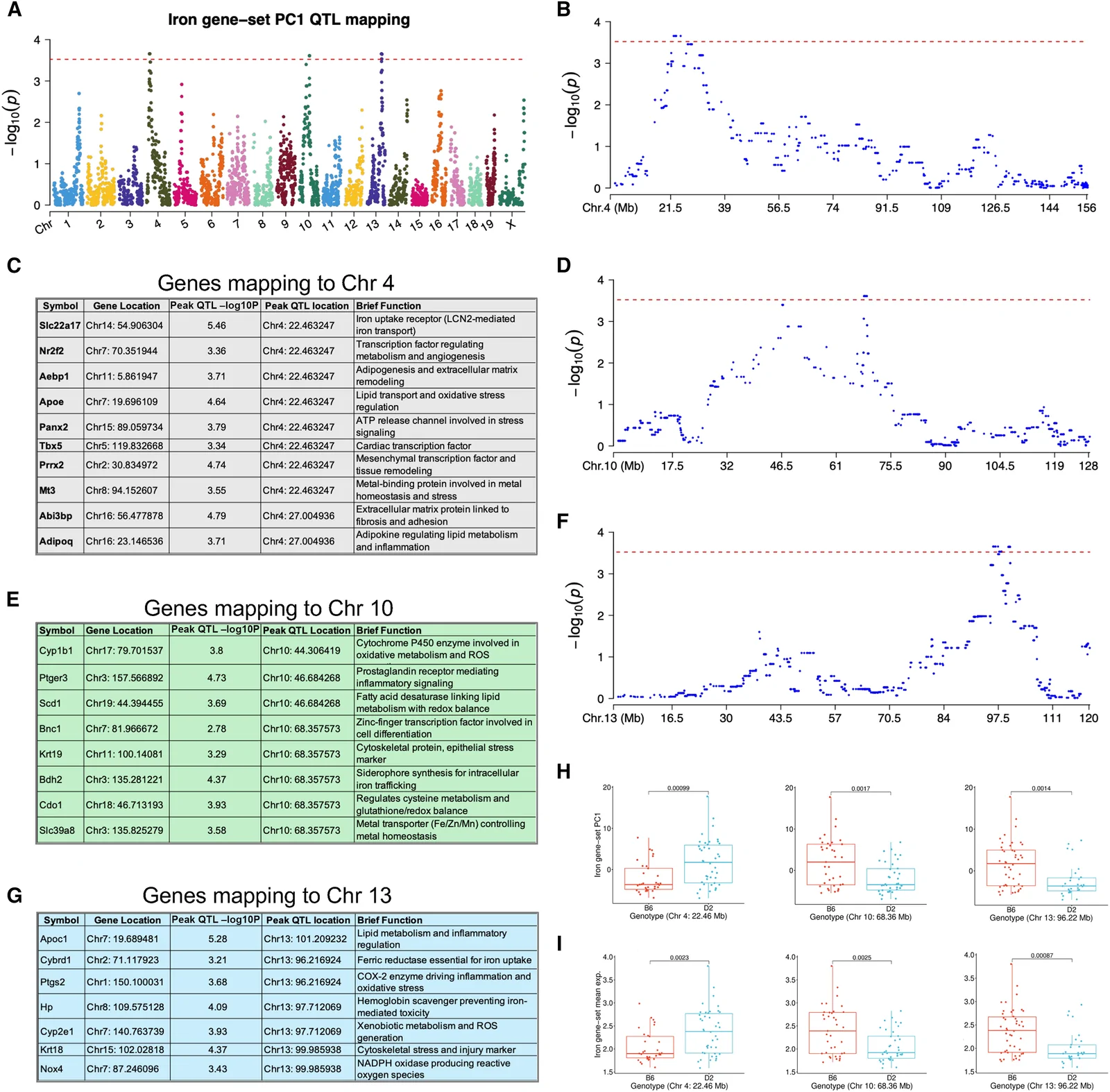
Genome-wide QTL mapping identifies three *trans*-eQTL hotspots regulating iron gene expression in BXD mouse heart (A) Manhattan plot of QTL mapping for iron gene-set PC1 derived from 38 iron-related yellow-module genes across 73 BXD strains. Each point represents a marker (−log_10_(*p*)); the dashed line indicates genome-wide significance (–log_10_*P* > 3.5). Significant peaks are observed on chromosomes 4, 10, and 13. (B, D, and F) Interval mapping plots for chromosome 4 (peak: 22.46 Mb, –log_10_*P* = 3.7), chromosome 10 (peak: 68.36 Mb, –log_10_*P* = 3.6), and chromosome 13 (peak: 96.22 Mb, –log_10_*P* = 3.7), representing three functional axes: iron-associated metabolic and inflammatory regulation, iron handling and redox regulation, and iron-responsive oxidative stress and detoxification, respectively. (C, E, and G) Tables listing genes mapping to each locus with genomic location, peak –log_10_*P*, QTL position, and brief functional annotation. (H) Boxplots of iron gene-set PC1 stratified by founder allele (B6 vs. D2) at each peak marker. Significant differences were observed at all loci (Chr4, *p* = 0.00099, D2 > B6; Chr10, *p* = 0.0017, B6 > D2; Chr13, *p* = 0.0014, B6 > D2; Wilcoxon rank-sum test), with individual strain values overlaid. (I) Boxplots showing the mean expression of the 38 iron-associated genes stratified by founder allele (B6 vs. D2) at each peak marker. Significant genotype-dependent differences were observed at all loci (Chr4, *p* = 0.0023, D2 > B6; Chr10, *p* = 0.0025, B6 > D2; Chr13, *p* = 0.00087, B6 > D2; Wilcoxon rank-sum test).

Genome-wide QTL mapping of the iron gene-set PC1 (of 38 genes based on BXD heart expression), which showed high variation across BXD strains (Figure S2) identified three genomic loci with a –log_10_*P* threshold >3.5 (*p* = 0.0003), with prominent peaks on chromosomes 4, 10, and 13 (Figure 4A). The strongest association was observed on chromosome 4 at 22.46 Mb (–log_10_*P* = 3.7) and chromosome 13 at 96.22 Mb (–log_10_*P* = 3.7), followed by a locus on chromosome 10 at 68.36 Mb (–log_10_*P* = 3.6). To validate this PCA-level finding, individual QTL mapping was performed for all 38 iron-related yellow-module genes. Notably, 25 of these genes mapped to one of the three identified loci, confirming their identity as major *trans*-eQTL hotspots and supporting their role as key regulatory hubs governing iron-related transcriptional variation in the BXD heart (Figure 4). The highest number of genes mapped to chromosome 4 (*n* = 10), followed by chromosome 10 (*n* = 8) and chromosome 13 (*n* = 7). Taken together, these results indicate that the iron gene-set PC1 is governed by a polygenic architecture with a limited number of dominant regulatory loci rather than a single major-effect locus, suggesting complex multi-layered genetic regulation involving distinct but potentially interacting biological pathways.

### Chromosome 4—Iron-associated metabolic and inflammatory regulatory axis

The chromosome 4 locus (peak: Chr4: 22.46–24.74 Mb) showed a well-defined QTL signal (Figure 4B) and regulated the largest number of iron-related genes among the three identified hotspots (*n* = 10; Figure 4C). All 10 regulated genes are physically located on different chromosomes, confirming the *trans*-eQTL nature of this locus. The regulated genes included *Slc22a17* (–log_10_*P* = 5.46), an iron uptake receptor mediating LCN2-dependent iron transport, and *Mt3* (–log_10_*P* = 3.55), a metal-binding protein involved in metal homeostasis and stress response, providing functional links to iron biology. The remaining genes were predominantly involved in lipid metabolism and inflammatory signaling—including *Apoe* (–log_10_*P* = 4.64), a regulator of lipid transport and oxidative stress, and *Adipoq* (–log_10_*P* = 3.71), an adipokine regulating lipid metabolism and inflammation—as well as extracellular matrix remodeling (*Abi3bp*, –log_10_*P* = 4.79; *Aebp1*, –log_10_*P* = 3.71), cardiac and mesenchymal transcriptional regulation (*Tbx5*, –log_10_*P* = 3.34; *Prrx2*, –log_10_*P* = 4.74; *Nr2f2*, –log_10_*P* = 3.36), and stress signaling (*Panx2*, –log_10_*P* = 3.79). The functional profile of this gene set identifies the chromosome 4 locus as an iron-associated metabolic and inflammatory regulatory axis, in which indirect links to iron biology are mediated through lipid metabolism, inflammatory signaling, and extracellular matrix remodeling. BXD strains carrying the D2 allele at the chromosome 4 peak marker (Chr4: 22.46 Mb) showed significantly higher iron gene-set PC1 values compared to B6 allele carriers (*p* = 0.00099; Figure 4H). Consistent with this finding, D2 allele carriers also exhibited significantly higher mean expression of the 38 iron-associated genes (*p* = 0.0023; Figure 4I), supporting increased coordinated expression of the iron-associated metabolic and inflammatory gene program.

### Chromosome 10—Iron handling and redox-regulatory axis

The chromosome 10 locus (peak: Chr10: 68.36–69.14 Mb) regulated eight genes, of which four showed direct or indirect functional relevance to iron biology (Figures 4D and 4E). The most directly iron-relevant genes regulated by this locus were *Bdh2* (–log_10_*P* = 4.37), involved in siderophore-mediated intracellular iron trafficking, and *Slc39a8* (–log_10_*P* = 3.58), a metal ion transporter controlling cellular iron, zinc, and manganese homeostasis. Two additional genes contributed indirectly through redox and oxidative mechanisms: *Cdo1* (–log_10_*P* = 3.93), regulating cysteine catabolism and glutathione-mediated redox balance relevant to ferroptosis, and *Cyp1b1* (–log_10_*P* = 3.80), a cytochrome P450 enzyme involved in oxidative metabolism and ROS generation. The remaining four genes—*Ptger3* (–log_10_*P* = 4.73), a prostaglandin receptor mediating inflammatory signaling; *Scd1* (–log_10_*P* = 3.69), a fatty acid desaturase linking lipid metabolism with redox balance; *Bnc1* (–log_10_*P* = 2.78), a zinc-finger transcription factor involved in cell differentiation; and *Krt19* (–log_10_*P* = 3.29), a cytoskeletal stress marker—were not directly iron-related but suggest broader regulatory influence of this locus beyond iron homeostasis per se. Notably, *Bdh2*, *Ptger3*, and *Slc39a8* are physically located on chromosome 3, confirming their *trans*-eQTL status and pointing to a shared upstream regulator at the chromosome 10 locus controlling intracellular iron handling and redox stress responses. With four iron-relevant genes representing 50% of its regulated gene set, the chromosome 10 locus represents the most iron-relevant hotspot identified. BXD strains carrying the B6 allele at the chromosome 10 peak marker (Chr10: 68.36 Mb) showed significantly higher iron gene-set PC1 values and higher mean expression of the 38 iron-associated genes than D2 allele carriers (*p* = 0.0017 and *p* = 0.0025, respectively; Figures 4H and 4I), indicating that the B6 allele at this locus is associated with higher expression of the iron-handling and redox-regulatory genes. This additive effect was also supported by individual gene-level QTL mapping.

### Chromosome 13—Iron-responsive oxidative stress and detoxification axis

The chromosome 13 locus (peak: Chr13, 96.22–97.43 Mb) regulated seven genes and harbored three iron homeostasis genes (Figures 4F and 4G). The most directly iron-relevant genes regulated by this locus were *Cybrd1* (–log_10_*P* = 3.21), a ferric reductase essential for reducing Fe^3+^ to Fe^2+^ for cellular iron uptake, and *Hp* (–log_10_*P* = 4.09), a hemoglobin scavenger protein that prevents iron-mediated toxicity by sequestering free hemoglobin. The co-regulation of *Cybrd1* and *Hp* from a shared genomic locus is particularly notable, as these genes represent complementary mechanisms of cardiac iron management—iron acquisition and iron detoxification, respectively—suggesting that the chromosome 13 locus coordinately regulates both the uptake and the safe handling of iron in the BXD heart. *Nox4* (–log_10_*P* = 3.43), an NADPH oxidase producing ROS, provided an additional indirect link to iron biology through ferroptosis-associated oxidative stress. The remaining genes regulated by this locus included *Apoc1* (–log_10_*P* = 5.28), a lipid metabolism and inflammatory regulation gene; *Ptgs2* (–log_10_*P* = 3.68), a COX-2 enzyme driving inflammation and oxidative stress; *Cyp2e1* (–log_10_*P* = 3.93), involved in xenobiotic metabolism and ROS generation; and *Krt18* (–log_10_*P* = 4.37), a cytoskeletal stress and injury marker. All seven genes are physically located on chromosomes other than chromosome 13, confirming the *trans*-eQTL nature of this hotspot. Although the chromosome 13 locus regulated fewer iron-relevant genes in absolute terms compared to chromosome 10 (three vs. four), its iron genes are more classically and specifically defined iron homeostasis regulators, identifying this locus as the qualitatively most iron-specific hotspot. BXD strains carrying the B6 allele at the chromosome 13 peak marker (Chr13: 96.22 Mb) showed significantly higher iron gene-set PC1 values (*p* = 0.0014; Figure 4H) and significantly higher mean expression of the 38 iron-associated genes (*p* = 0.00087; Figure 4I) than D2 allele carriers, supporting increased coordinated expression of iron-responsive oxidative stress and detoxification genes, which is also consistent with the additive effects observed for individual iron genes regulated by this locus.

### Opposing allelic effects reveal distinct regulatory mechanisms across loci

Allelic effect analysis using the Wilcoxon rank-sum test revealed a mixed but interpretable directional pattern across the three QTL loci (Figure 4H). At the chromosome 4 locus, D2 allele carriers showed significantly higher iron gene-set PC1 values compared to B6 carriers (*p* = 0.00099), indicating that D2-derived variants at this locus are associated with higher expression of iron-associated metabolic and inflammatory gene expression. In contrast, at both the chromosome 10 and 13 loci, B6 allele carriers showed significantly higher PC1 values compared to D2 carriers (*p* = 0.0017 and *p* = 0.0014, respectively), indicating that B6-derived variants at these loci are associated with higher expression of iron-handling, redox-regulatory, and iron-responsive oxidative stress and detoxification genes. The same directional pattern was observed when the mRNA expression of the iron gene set was compared between genotypes (Figure 4I). This opposing allelic directionality suggests that the genetic architecture of cardiac iron gene expression involves founder-specific regulatory variants acting through mechanistically distinct transcriptional pathways. The divergent allelic effects across loci further support the polygenic nature of cardiac iron gene regulation in the BXD panel and highlight the complexity of genetic interactions underlying iron homeostasis in the heart.

### Identification of candidate genes affecting the expression of iron gene set and underlying heart functions

**Figure 5:**
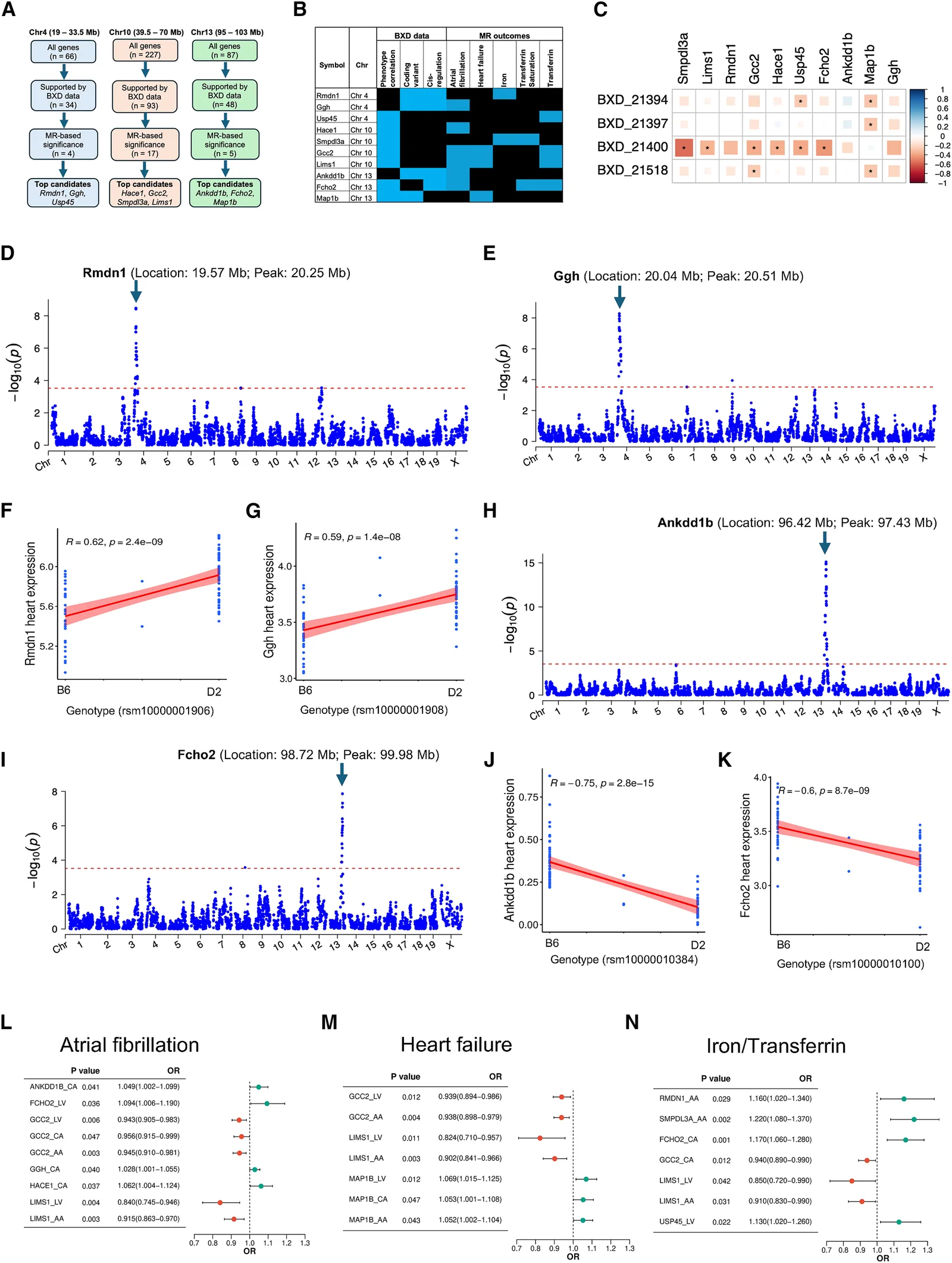
Prioritization of candidate genes using systems genetics and causal analysis (A) Workflow for candidate gene prioritization from QTL intervals using BXD-based evidence (expression-trait correlation, *cis*-eQTLs, coding variants) followed by Mendelian randomization (MR) filtering across loci on chromosomes 4, 10, and 13. (B) Heatmap summarizing genetic and functional support for prioritized candidate genes, integrating BXD data and human MR outcomes. Blue box indicates supporting evidence; black box indicates no evidence. (C) Correlation heatmap showing relationships between candidate gene expression and phenotypes across BXD strains. (D, E, H, and I) Genome-wide QTL mapping plots for *cis*-regulated candidate genes (*Rmdn1*, *Ggh*, *Ankdd1b*, and *Fcho2*). –log_10_*P* values are plotted across genomic positions; the red dashed line indicates the genome-wide significance threshold (–log_10_*P* > 3.5), with arrows marking peak loci. (F, G, J, and K) Scatterplots showing genotype-dependent expression (*cis*-eQTL effects) for the same genes at their respective peak markers, with linear regression fits, Pearson’s correlation coefficients (R), and *p* values indicated. (L–N) Mendelian randomization results in human datasets. Forest plots display odds ratios (ORs) with 95% confidence intervals across tissues—left ventricle (LV), coronary artery (CA), and atrial appendage (AA)—highlighting significant associations with atrial fibrillation, heart failure, and iron/transferrin-related traits.

Candidate genes were identified within the 1.5-LOD confidence intervals of each QTL (Figure 5A) and subjected to a stepwise prioritization strategy. Across loci on chromosomes 4 (19–33.5 Mb), 10 (39.5–70 Mb), and 13 (95–103 Mb), we identified 66, 227, and 87 protein-coding genes, respectively. Integration of BXD-based evidence—including expression-trait correlations, coding sequence variants between the parental strains (B6 and D2) and *cis*-eQTL support—reduced these to 34, 93, and 48 genes, respectively. Subsequent filtering using MR analysis further refined the candidate lists to four, 17, and five genes with significant associations (Table 1), of which seven genes remained significant following Benjamini-Hochberg multiple testing correction (FDR-adjusted *p* <0.10). None of the prioritized candidate genes showed significant heterogeneity, and MR-Egger intercept analysis did not yield informative results because the reported significant associations were based on single-instrument (Wald ratio) analyses (Data S5). We finally identified a set of top candidates from each locus that were supported by genetic evidence in BXD mice, causal inference from human MR analyses and functional significance in iron homeostasis in heart: *Rmdn1*, *Ggh*, and *Usp45* for the chromosome 4 locus; *Hace1*, *Gcc2*, *Smpdl3a*, and *Lims1* for the chromosome 10 locus; and *Ankdd1b*, *Fcho2*, and *Map1b* for the chromosome 13 locus (Figure 5B), highlighting their potential roles in modulating iron levels in heart and the underlying function.

**Table 1:** Candidate genes supported by BXD data and showing causal associations in human cardiac tissues

Symbol	Chr	Heart expression	Phenotype correlation	Coding variant (B6 vs. D2)	*Cis* regulation	Outcome
*Rmdn1*	Chr 4	5.76	–	✓	✓	IR
*Ggh*	Chr 4	3.61	–	✓	✓	AF
*Usp45*	Chr 4	2.62	✓	–	–	TF
*Lyrm2*	Chr 4	4.90	✓	–	–	IR
*Rpf2*	Chr 10	3.80	✓	–	–	IR, TS
*Cdk19*	Chr 10	3.72	✓	–	–	AF
*Mettl24*	Chr 10	2.14	✓	–	–	TR
*Fig4*	Chr 10	5.45	✓	–	–	AF, HF
*Hace1*	Chr 10	1.54	✓	–	–	AF
*Grik2*	Chr 10	0.32	✓	–	–	AF, IR, TS
*Ascc3*	Chr 10	1.52	✓	–	–	HF
*Vgll2*	Chr 10	1.22	✓	–	–	AF
*Dcbld1*	Chr 10	1.98	✓	–	–	AF
*Gopc*	Chr 10	2.84	✓	–	–	AF, HF
*Hsf2*	Chr 10	2.40	✓	–	–	AF
*Smpdl3a*	Chr 10	6.20	✓	–	–	IR, TS
*Gcc2*	Chr 10	2.70	✓	–	–	AF, HF, TR
*Lims1*	Chr 10	5.97	✓	–	–	AF, HF, TR
*Ccdc138*	Chr 10	0.95	✓	–	–	AF
*Tspan15*	Chr 10	2.73	✓	–	–	TS
*Herc4*	Chr 10	3.35	✓	–	–	AF
*F2rl2*	Chr 13	0.001	–	✓	–	AF, TS
*Ankdd1b*	Chr 13	0.27	–	✓	✓	AF
*Btf3*	Chr 13	7.27	✓	–	–	HF
*Fcho2*	Chr 13	3.41	✓	–	✓	AF, TS, TR
*Map1b*	Chr 13	2.02	✓	✓	–	HF

AF, atrial fibrillation (id:ebi-a-GCST006414); HF, heart failure (id:ebi-a-GCST009541); IR, iron (id:ieu-a-1049); TS, transferrin saturation (id:ieu-a-1051); TR, transferrin (id:ieu-a-1052).

### *Rmdn1*, *Ggh*, and *Usp45* are the top candidates from chromosome 4 locus

We identified *Rmdn1*, *Ggh*, and *Usp45* as top candidates at the chromosome 4 locus based on integration of BXD genetics and human causal inference (Figures 5A and 5B). *Usp45* showed consistent negative correlations across multiple BXD iron-related traits (r ≈ −0.26 to −0.38, *p* < 0.05) (Figure 5C), despite lacking strong positional support at the QTL peak, suggesting a regulatory role. In contrast, *Rmdn1* and *Ggh* map close to the primary QTL peak (∼20.5 Mb) with robust linkage signals (–log_10_*P* ≈ 8.5 and 8.3, respectively) and harbor functional coding variants. Both genes exhibit strong *cis*-eQTL effects, with BXD strains carrying the D2 allele showing significantly higher expression compared to B6 (Figures 5D–5G). These associations were consistent across multiple independent BXD datasets, supporting robustness of the locus.

Expression analysis across multiple human tissues showed that *RMDN1* and *USP45* are broadly expressed, including in cardiovascular and metabolically active tissues, whereas *GGH* displayed more variable expression but remained detectable in several tissues. Single-cell expression analysis further revealed that all three genes are expressed across multiple cardiac cell types, with *RMDN1* and *USP45* showing relatively higher expression in cardiomyocytes compared to other cell populations. These patterns support the potential relevance of these genes in cardiac and systemic metabolic processes (Figures S3 and S4).

MR analysis further supported this region, indicating a potential role in increased iron levels for *RMDN1* in atrial tissue (odds ratio [OR] = 1.16, *p* = 0.029) and transferrin levels for *USP45* in LV tissue (OR = 1.13, *p* = 0.022). These genes likely promote iron accumulation or systemic availability (Figure 5N). Functionally, these genes converge on pathways relevant to iron homeostasis: *Rmdn1* regulates mitochondria-ER contact sites important for mitochondrial iron utilization, *Ggh* controls folate metabolism required for heme biosynthesis, and *Usp45* encodes a deubiquitinating enzyme implicated in protection against genomic stress. Based on its strong positional evidence, functional coding variation, *cis*-eQTL regulation, and support from human MR analysis, *Rmdn1* was considered the high-confidence candidate at the chromosome 4 locus, whereas *Ggh* and *Usp45* were considered exploratory candidates.

### *Hace1*, *Gcc2*, *Smpdl3a*, and *Lims1* are the top candidates from chromosome 10 locus

We prioritized *Hace1*, *Gcc2*, *Smpdl3a*, and *Lims1* as candidate regulators on the chromosome 10 locus (Figures 5A and 5B). Although all four candidates were supported by only phenotype correlation in BXD mice, subsequent functional and human MR analyses provided varying levels of additional support. Notably, *Hace1* is a curated iron-related gene, providing a direct link to the study’s central focus. Functionally, *Gcc2* regulates vesicle trafficking and transferrin receptor recycling, *Hace1* modulates oxidative stress responses, *Smpdl3a* influences lipid metabolism and ferroptosis susceptibility, and *Lims1* supports cell adhesion and myocardial structural integrity. Expression-trait analysis revealed consistent negative correlations for all four genes, suggesting suppressive effects on the BXD iron phenotype (Figure 5C). Among these, *Smpdl3a* exhibited the strongest association (r = −0.55, *p* = 0.0011), followed by reproducible effects for *Gcc2* (r ≈ −0.26 to −0.36, *p* < 0.05). The clinical relevance of this locus was further supported by *LIMS1*, which showed significant downregulation in human cardiomyopathy dataset (FC ≈ −1.57, FDR < 0.01).

Tissue-level and single-cell expression analyses demonstrated that these genes are broadly expressed across metabolically active tissues, with detectable expression in cardiomyocytes (Figures S3 and S4). These patterns support their potential involvement in both local cardiac processes and broader systemic regulation. Human MR analysis further supported this locus, with *GCC2* associated with reduced risk of atrial fibrillation (left ventricle, OR = 0.94, *p* = 0.006; coronary artery, OR = 0.96, *p* = 0.047; atrial appendages, OR = 0.95, *p* = 0.003) and heart failure (left ventricle, OR = 0.94, *p* = 0.012; atrial appendages, OR = 0.94, *p* = 0.004), while *HACE1* showed association with increased risk of atrial fibrillation (OR = 1.06, *p* = 0.037). *LIMS1* was also associated with reduced risk of atrial fibrillation (left ventricle, OR = 0.84, *p* = 0.004; atrial appendages, OR = 0.91, *p* = 0.003) and heart failure (left ventricle, OR = 0.824, *p* = 0.011; atrial appendages, OR = 0.90, *p* = 0.003) (Figures 5L–5M). Interestingly, *GCC2*, *SMPDL3A*, and *LIMS1* were significantly associated with iron/transferrin-related outcomes. *SMPDL3A* showed strong causal associations with both increased serum iron (OR = 1.215, *p* = 0.0017) and transferrin saturation (OR = 1.222, *p* = 0.0014) in atrial appendage tissue, whereas *GCC2* and *LIMS1*, which are highly protective against heart failure and atrial fibrillation, showed negative associations (OR < 1) with transferrin levels, further supporting their role as “buffers” that prevent iron-mediated toxicity (Figure 5N). Based on the convergence of its functional relevance to iron homeostasis and support from multiple human MR outcomes, *Gcc2* emerged as the high-confidence candidate at the chromosome 10 locus. Together, these findings suggest that the chromosome 10 locus may act through coordinated multi-gene effects integrating iron handling, stress response, and structural maintenance in cardiac tissue.

### *Ankdd1b*, *Fcho2*, and *Map1b* are the top candidates from chromosome 13 locus

Integration of positional mapping, correlation analysis, transcriptional filtering, and human causal inference prioritized *Ankdd1b*, *Fcho2*, and *Map1b* as top candidate genes at the chromosome 13 locus (Figure 5B). *Fcho2* regulates clathrin-mediated endocytosis, a pathway involved in transferrin-bound iron uptake, whereas *Ankdd1b* contains ankyrin repeat and death domains consistent with roles in protein interaction and stress signaling, and *Map1b* regulates microtubule dynamics required for intracellular transport. Both *Ankdd1b* and *Map1b* harbor non-synonymous coding variants between the founder strains.

Expression-trait analysis in BXD heart data revealed significant inverse associations, with *Fcho2* showing a robust correlation (R = −0.44, *p* = 0.01) and *Map1b* demonstrating consistent negative correlations across multiple datasets, supporting its contribution to the locus (Figure 5C). Among all loci, the chromosome 13 interval showed the strongest genetic signal, with *Ankdd1b* exhibiting a prominent *cis*-eQTL peak (–log_10_*P* = 15.1) and *Fcho2* with a highly significant *cis*-eQTL (–log_10_*P* = 7.9) (Figures 5H and 5I). BXD strains carrying the B6 genotype displayed higher expression of both genes compared to those with the D2 genotype at the peak (Figures 5J and 5K).

Human tissue-level and single-cell expression analyses (Figures S3 and S4) confirmed the cardiac relevance of these genes. *FCHO2* is broadly expressed across cardiac tissues, whereas *ANKDD1B* shows lower baseline expression but strong genotype-dependent regulation, consistent with a regulatory role. Human MR analysis further supported this locus, with *ANKDD1B* associated with atrial fibrillation (OR = 1.049, *p* = 0.041), *FCHO2* associated with atrial fibrillation (OR = 1.094, *p* = 0.036) and iron-related traits, and *MAP1B* showing associations with heart failure outcomes (OR ≈ 1.05–1.07, *p* < 0.05) across multiple cardiac tissues (Figures 5L–5N). Based on the convergence of multiple lines of evidence in BXD mice and associations with multiple outcomes in the human MR analysis, *Fcho2* was considered a high-confidence candidate at the chromosome 13 locus. In contrast, *Ankdd1b* and *Map1b*, each supported by a single MR outcome, were considered exploratory candidates. Together, these findings suggest that the chromosome 13 locus contributes to cardiac phenotype regulation through coordinated effects on endocytosis, cellular signaling, and cytoskeletal organization.

### Candidate gene-correlated transcripts link to intracellular transport and metabolic regulation in cardiac tissue

**Figure 6:**
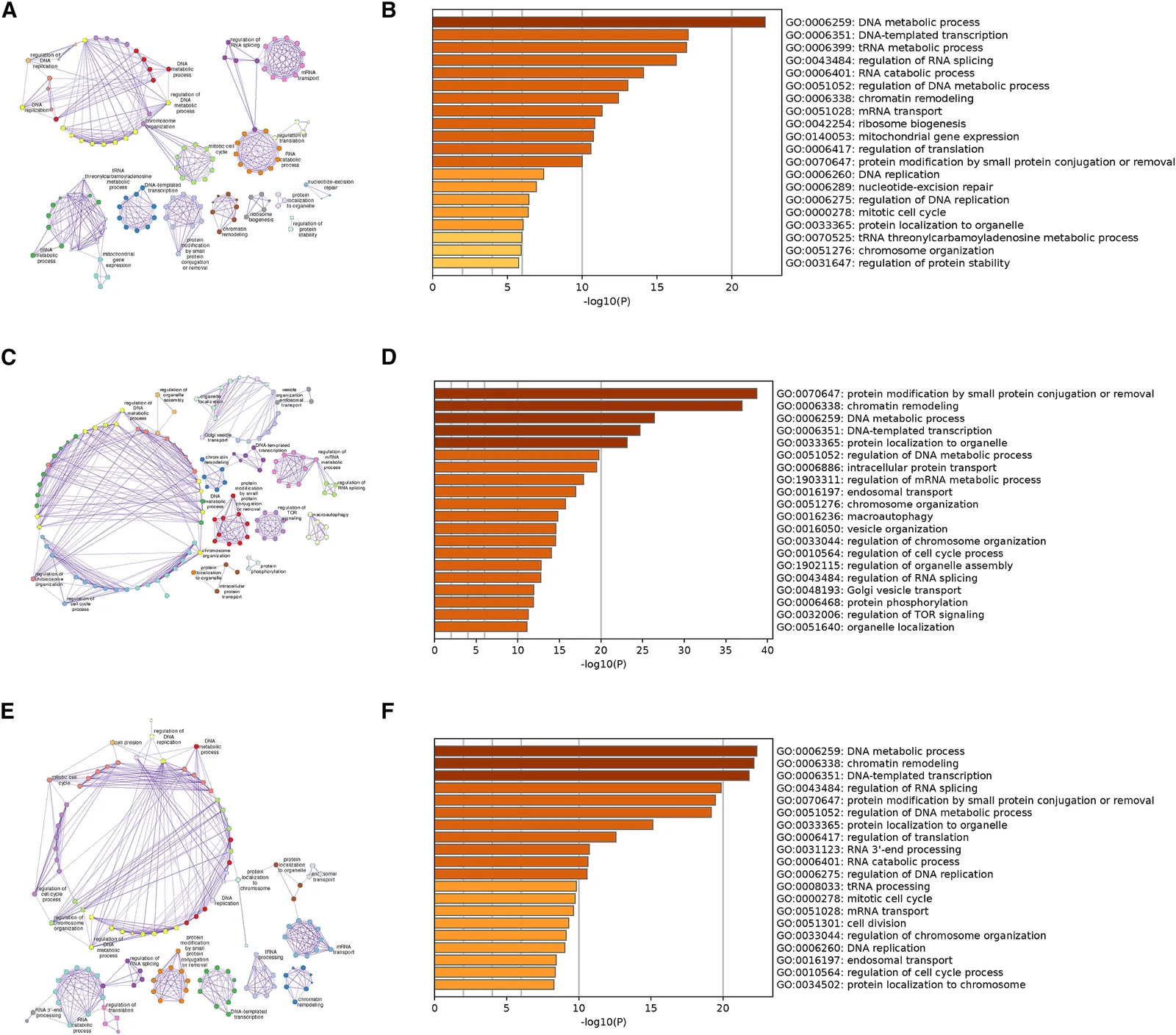
Network-based functional characterization of candidate gene-correlated transcripts (A, C, and E) Functional enrichment networks generated using Metascape from the top 2,000 genes most strongly correlated with *Rmdn1* (A), *Gcc2* (C), and *Fcho2* (E) expression in the BXD heart dataset. Each node represents an enriched biological process, and edges indicate pathway similarity based on shared genes. Nodes are grouped into clusters based on functional similarity, with representative terms shown for each cluster. (B, D, and F) Bar plots showing the enriched Gene Ontology (GO) biological processes for *Rmdn1* (B), *Gcc2* (D), and *Fcho2* (F), ranked by statistical significance (−log10(*p*)).

Based on BXD evidence and causal significance in human MR analysis, we identified *Rmdn1*, *Gcc2*, and *Fcho2* as the high-confidence candidates from Chr 4, 10, and 13 loci, respectively. To define the biological programs associated with these genes, we analyzed the top 2,000 transcripts that were significantly correlated with these genes in the BXD heart dataset and performed GO enrichment and network analysis (Figure 6). Enriched terms were visualized as network clusters (Figures 6A, 6C, and 6E) and ranked by significance (−log10(*p*); Figures 6B, 6D, and 6F), enabling assessment of both functional enrichment and network topology.

*Rmdn1*-correlated genes were enriched for RNA metabolism, translation, and protein homeostasis pathways, including mRNA transport, ribosome biogenesis, and RNA catabolic processes, along with mitochondrial gene expression and tRNA metabolism. The corresponding network displayed a dispersed architecture composed of multiple moderately connected clusters. A prominent, highly interconnected cluster related to DNA metabolic and cell cycle processes was also observed, likely reflecting broadly co-regulated cellular programs. Overall, these results suggest that *Rmdn1* is associated with mitochondrial function and general cellular homeostasis rather than a single specialized pathway (Figures 6A, 6B, and S5).

*Gcc2* exhibited a distinct and highly coherent enrichment profile dominated by vesicle-mediated transport pathways, including endosomal transport, Golgi vesicle trafficking, and intracellular protein localization. Consistent with this, the network formed a compact and densely interconnected structure, with trafficking-related clusters comprising a central module characterized by high gene overlap. Additional enrichment of protein modification and signaling pathways suggests coordinated regulation of trafficking machinery. Together, these findings support a central role for *Gcc2* in intracellular transport and membrane trafficking processes (Figures 6C, 6D, and S6).

The genes correlated with *Fcho2* were enriched for protein localization to organelles and endosomal transport, consistent with its role in clathrin-mediated endocytosis, along with pathways related to protein ubiquitination and intracellular protein transport. The network showed an intermediate topology, with smaller trafficking-related clusters embedded within a broader structure enriched for RNA processing and DNA metabolic pathways. These broader signals likely reflect general co-expression patterns, whereas the more localized clusters highlight *Fcho2*-specific functions in vesicle formation and cargo internalization (Figures 6E, 6F, and S7).

Collectively, these analyses reveal distinct yet complementary functional modules across candidate genes. While *Gcc2* is associated with a tightly coordinated vesicle trafficking network, *Fcho2* contributes to endocytic initiation and protein localization, and *Rmdn1* is linked to mitochondrial function and cellular homeostasis. These findings support a coordinated model in which candidate genes act at different stages of intracellular transport and metabolic regulation in cardiac tissue.

## Discussion

Iron homeostasis is critical for maintaining cardiac function due to its central role in mitochondrial energy production, redox balance, and cellular metabolism. Disruption of iron balance—whether through deficiency or overload—has been strongly associated with cardiac dysfunction, including impaired contractility, pathological remodeling, and increased susceptibility to arrhythmias.^48^^,^^49^^,^^50^ Despite extensive knowledge of systemic iron regulation, the genetic and molecular mechanisms governing iron levels within cardiac tissue remain incompletely understood. In this study, we leveraged a systems genetics approach in BXD mice to dissect the genetic architecture of cardiac iron homeostasis, integrating QTL mapping, transcriptomic analysis, and cross-species validation.

The observed variation in cardiac iron across genetically diverse BXD strains highlights the importance of inherited factors in shaping myocardial iron balance, but, more importantly, it provides insight into how iron may influence cardiac physiology over time. Iron appears to modulate specific functional domains of the heart, with distinct structural and electrical consequences.^14^^,^^51^ The pattern observed suggests that altered iron availability may initially impact myocardial growth and energy balance, consistent with its central role in mitochondrial function and cellular metabolism.^14^^,^^52^ Over time, however, the effects appear to extend to the electrophysiological system, potentially through mechanisms involving oxidative stress, ion channel modulation, or disruption of intracellular signaling pathways.^53^^,^^54^ Collectively, these findings support a model in which cardiac iron is an active determinant of cardiac remodeling and electrical stability.

We constructed a co-expression network using BXD heart transcriptomic data and identified a gene module that was significantly associated with cardiac iron levels and overlapped with curated set of iron-related genes. Functional enrichment analysis indicates that this module represents a coordinated transcriptional network linked to iron-dependent metabolic, cardiovascular, and regulatory processes. Enrichment of cAMP signaling and circadian entrainment pathways suggests integration of systemic and temporal regulation of iron homeostasis, as cAMP signaling modulates hepcidin expression, while circadian entrainment has been linked to systemic metabolic regulation in tissues with high iron demand, suggesting that the co-expression module may coordinate transcriptional responses to fluctuating cardiac iron availability across temporal axes.^6^^,^^55^^,^^56^ The presence of dopaminergic and glutamatergic synapse pathways likely reflects the fundamental requirement of iron for mitochondrial function and redox balance across tissues, including the heart.^57^^,^^58^ In addition, enrichment for cardiovascular and metabolic phenotypes supports the interpretation that this module captures transcriptional responses to disrupted iron homeostasis, as both iron deficiency and overload impair mitochondrial energetics and promote oxidative stress.^4^^,^^17^ The enrichment of oxidative stress and ferroptosis pathways further implicates iron-driven redox imbalance as a key mechanism linking cardiac iron levels to tissue dysfunction.^19^^,^^59^ Notably, endocardial cells were the most significantly enriched cell type, consistent with their role in iron handling and sensitivity to oxidative stress, and the negative correlation between this module and BXD cardiac iron levels suggests an inverse relationship between network activity and cardiac iron burden,^60^^,^^61^ although the direction of this association cannot be determined from the cross-sectional data. Importantly, genes within this module are associated with heart failure and overlap with human cardiomyopathy-related expression signatures, aligning with clinical evidence that iron dysregulation contributes to disease progression.^62^^,^^63^ However, as the human dataset represents DCM rather than specifically iron-overload cardiomyopathy, this overlap should be interpreted as evidence of broader relevance to cardiac disease.

Furthermore, overlap between the yellow module and the curated iron gene set identified 38 genes shared between the two, which exhibited high intramodular connectivity, significant correlations with BXD iron phenotypes, and strain-specific expression patterns across the BXD population, indicative of strong genetic regulation. To identify the upstream genetic drivers of this iron-associated transcriptional network, we performed QTL mapping on the first principal component summarizing the joint expression of the 38 genes, which revealed significant peaks on chromosomes 4, 10, and 13. This multi-chromosomal architecture was further corroborated by individual QTL mapping of each of the 38 genes, reinforcing the robustness of these loci as regulators of the module’s iron-related transcriptional signature. Of the 38 module iron genes, 25 mapped to one of the three identified chromosomal loci, confirming chromosomes 4, 10, and 13 as major *trans*-eQTL hotspots governing the transcriptional regulation of cardiac iron homeostasis within the co-expression module. Furthermore, the opposing allelic effects across the three *trans*-eQTL hotspots suggest that genetic regulation of cardiac iron in the BXD panel is driven by founder-specific variants with distinct functions, indicating that different genetic backgrounds influence cardiac iron regulation through separate biological mechanisms.^64^

The chromosome 10 locus is consistent with prior BXD-based QTL analyses of iron homeostasis traits, which identified a significant marker at approximately 64 Mb on chromosome 10 associated with spleen iron concentration under dietary iron manipulation.^65^ The chromosome 13 peak is particularly noteworthy, as the murine *Hfe* gene—the principal genetic determinant of hereditary hemochromatosis and a master regulator of systemic iron absorption—maps to chromosome 13 in mice, having been translocated from its syntenic MHC-proximal position during mouse genome evolution.^66^ Collectively, the polygenic architecture implied by regulatory peaks spanning three chromosomes is consistent with the well-established view that tissue iron levels are governed by multiple independent genomic loci acting in concert^67^^,^^68^ and positions the selected co-expression module as a transcriptional readout of this distributed genetic control of cardiac iron homeostasis.

Next, we identified the genetic regulators responsible for coordinating the expression of the iron gene set within the co-expression module. The 1.5-LOD support intervals across the three chromosomal loci collectively harbored more than 350 candidate genes. Through systematic integration of BXD expression data, functional annotation, and human disease relevance, we prioritized 10 candidate regulators—three each from chromosomes 4 and 13, and four from chromosome 10. The high-confidence genes from chromosomes 4, 10, and 13 were *Rmdn1*, *Gcc2*, and *Fcho2*, respectively, each implicating a distinct step in the cellular iron trafficking axis.

*Rmdn1* (regulator of microtubule dynamics 1) encodes a protein associated with microtubules and mitochondria and has been implicated in cytoskeletal organization and intracellular trafficking processes. Although its direct role in iron metabolism has not been fully characterized, microtubule dynamics are critical for the directed transport of endosomes and vesicles within cells, including those involved in transferrin-mediated iron uptake.^69^^,^^70^ Following internalization, iron is trafficked through endosomal compartments and delivered to mitochondria, the primary site of iron utilization for heme synthesis and iron-sulfur cluster assembly.^3^^,^^71^ Thus, *Rmdn1* may influence intracellular iron distribution by regulating microtubule-dependent vesicular trafficking and mitochondria-associated processes.^72^ Microtubule-dependent organelle positioning is furthermore critical for the structural integrity of mitochondria-ER contact sites (MAMs), which regulate not only calcium signaling but also lipid transfer and metabolic coordination in cardiomyocytes.^73^^,^^74^ The association of *Rmdn1* with the mitochondrial compartment and its co-expression with iron homeostasis genes within the yellow module suggests a role in coordinating the cytoskeletal infrastructure that supports iron delivery and utilization at the mitochondrial interface.

*Gcc2* encodes GCC185, a large coiled-coil tethering protein of the trans-Golgi network (TGN) that functions as a *Rab9* effector required for the retrograde transport of vesicles from late endosomes back to the TGN. This pathway is essential for the recycling of mannose-6-phosphate receptors and other sorting receptors; critically, it also maintains the membrane availability and recycling efficiency of TfR1, which, after iron delivery in the acidified endosome, must be returned to the cell surface for subsequent rounds of iron uptake.^75^^,^^76^^,^^77^ Loss of GCC185 disrupts Golgi architecture, impairs endosome-to-TGN transport, and leads to mis-sorting and enhanced degradation of recycling receptors, which would be expected to reduce steady-state TfR1 surface availability and thereby constrain cellular iron import capacity.^76^^,^^78^

*Fcho2* (FCH and mu domain-containing endocytic adaptor 2) encodes an F-BAR domain protein that functions as an early organizer of clathrin-coated pit assembly at the plasma membrane. Through its membrane-bending activity and interaction with the AP2 adaptor complex, FCHO2 nucleates clathrin-coated pit formation and directly regulates the endocytosis of transferrin receptor (TfR1)—the principal entry point for iron-loaded transferrin into cardiomyocytes, where TfR1-mediated iron uptake is essential for maintaining mitochondrial function and preventing cardiomyopathy.^79^^,^^80^^,^^81^ Depletion of FCHO2 in cell models reduces the number of clathrin-coated structures at the plasma membrane and impairs transferrin receptor clustering at coated pits, indicating that FCHO2 regulates the efficiency of coated pit organization required for transferrin receptor-mediated iron uptake.^82^ Together, *Fcho2*, *Gcc2*, and *Rmdn1* delineate a coherent intracellular framework—from iron entry at the plasma membrane, through endosomal recycling and Golgi sorting, to cytoskeletal support of mitochondrial iron utilization—that collectively defines how genetic variation at the three *trans*-eQTL hotspots may translate into altered cardiac iron balance and the transcriptional phenotype captured by the co-expression network.

Several limitations should be acknowledged. First, the QTL mapping captures genetic control of iron-related gene expression rather than iron levels directly, and the phenotype-transcriptome associations reported here are correlational and require functional validation. Second, principal component (PC1) was calculated using strain-level transcriptomic data and therefore represents a summary measure of coordinated transcriptional variation within the iron-associated gene set. Although explicit adjustment for potential covariates such as age and sex was not possible, the biological relevance of the identified loci is supported by concordant genotype-dependent differences in the mean expression of the iron-associated gene set, together with consistent additive effects observed in individual gene-level QTL analyses. Finally, generalizability beyond the BXD system remains to be established. Future work should focus on cardiomyocyte-specific characterization of the candidate regulators and longitudinal studies linking cardiac iron accumulation to functional cardiac outcomes over time.

Overall, our study advances a systems-level understanding of cardiac iron homeostasis, demonstrating that myocardial iron levels vary substantially across BXD strains under heritable genetic control and carry measurable consequences for cardiac structure and electrophysiology. The yellow module, negatively correlated with cardiac iron, anchored in endocardial biology, and conserved in human cardiomyopathy gene sets, represents a transcriptional network bridging iron dysregulation and cardiac pathology. Candidate *trans*-eQTL regulators *Fcho2*, *Gcc2*, and *Rmdn1* delineate sequential steps in intracellular iron trafficking—from plasma membrane endocytosis through endosomal recycling to mitochondrial iron utilization—providing a mechanistic foundation for how genetic variation shapes the transcriptional regulation of cardiac iron-related processes.

In summary, this study employs a systems genetics framework in BXD mice to dissect the transcriptional regulation of cardiac iron homeostasis. Weighted gene co-expression analysis of the BXD heart transcriptome identified a gene module negatively correlated with cardiac iron levels, enriched for endocardial cell biology and metabolic regulation, and significantly overlapping with human cardiomyopathy gene expression signatures. QTL mapping of the principal component of 38 coexpressed iron-module genes, corroborated by individual gene mapping, identified regulatory hotspots on chromosomes 4, 10, and 13, implicating *Rmdn1*, *Gcc2*, and *Fcho2* as the high-confidence candidate *trans*-regulators of this network. Phenotypic correlations further linked elevated cardiac iron to reduced ventricular mass, increased ventricular ectopy, and prolonged atrioventricular conduction, consistent with an iron-overload cardiomyopathy signature. Together, these findings provide a comprehensive systems-level map of cardiac iron gene regulation and a molecular framework for understanding how disruption of iron-related transcriptional networks contributes to structural and electrical cardiac dysfunction.

## Data and code availability

The datasets generated during and/or analyzed during the current study are available in GeneNetwork (https://info.genenetwork.org/infofile/source.php?GN_AccesionId=1028). The scripts used for major analysis are provided as supplemental information.

## Acknowledgments

We thank Drs. Buyan-Ochir Orgil and Wenyuan Zhao from University of Tennessee Health Science Center for generating the data and Arthur Centeno from University of Tennessee Health Science Center for making data available on GeneNetwork (https://www.genenetwork.org).

This work was supported by 10.13039/100000002National Institutes of Health grants R01 HL151438 (L.L. and E.P.), Shenzhen Medical Research Fund B2402041 (Q.Z.) and the Special Funding for the “Case-by-Case Introduction of Top Talent (Teams)” Program in Yantai.

## Author contributions

A.K.B., formal analysis, methodology, visualization, data curation, and writing – original draft; Q.G., formal analysis, data curation, validation, visualization, and writing – original draft; R.K., formal analysis and writing – review and editing; X.L., resources and writing – review and editing; A.S.-D., data curation and writing – review and editing; W.Z., investigation, formal analysis, writing – review and editing; EP, resources, investigation, and writing – review and editing; B.C.J., investigation, resources, and writing – review and editing; Q.Z., funding acquisition and writing – review and editing; D.W., funding acquisition and writing – review and editing; L.L., conceptualization, project administration, methodology, supervision, funding acquisition, and writing – review and editing. All authors read and approved the final version of the manuscript.

## Declaration of interests

The authors declare no competing interests.

## Web resources

Cytoscape: http://cytoscape.org/
DisGeNET: https://disgenet.com/
Enrichr: https://maayanlab.cloud/Enrichr
Ensembl: https://www.ensembl.org/
FerrDB: https://www.zhounan.org/ferrdb/
GeneCards: https://www.genecards.org/
GeneNetwork: https://genenetwork.org/
GO: http://geneontology.org/
GTEx: https://gtexportal.org/
Human Protein Atlas: https://www.proteinatlas.org/
KEGG: https://www.genome.jp/kegg/
Metascape: https://metascape.org/
WebGestalt: https://www.webgestalt.org/

## Supplemental information

Document S1. Figures S1–S7, Table S1, and Text S1

Data S1. Correlation of cardiac iron levels with echocardiographic, hemodynamic, and electrocardiographic phenotypes

Data S2. Details of WGCNA co-expression modules

Data S3. Functional analysis of yellow module genes

Data S4. Correlation between module iron genes and BXD cardiac iron levels

Data S5. Mendelian randomization analysis results

Document S2. Article plus supplemental information
