# Supplementary material for: Genetic dissection of cardiac iron regulation using transcriptome network analysis and systems genetics in BXD mice
Source: HGG Adv. 2026 Sep 1;7(4):100668. doi: 10.1016/j.xhgg.2026.100668 (PMC13594880; doi:10.1016/j.xhgg.2026.100668)
Supplement: Supplementary file 1 [file mmc1.pdf]

HGGA, Volume 7

Supplemental information

Genetic dissection of cardiac iron regulation using transcriptome network analysis and systems genetics in BXD mice

Akhilesh K. Bajpai, Qingqing Gu, Rakesh Kumar, Xiangtang Li, Athena Starlard-Davenport, Wenjing Zhang, Enkhsaikhan Purevjav, Byron C. Jones, Qingyin Zheng, Dong Wang, and Lu Lu

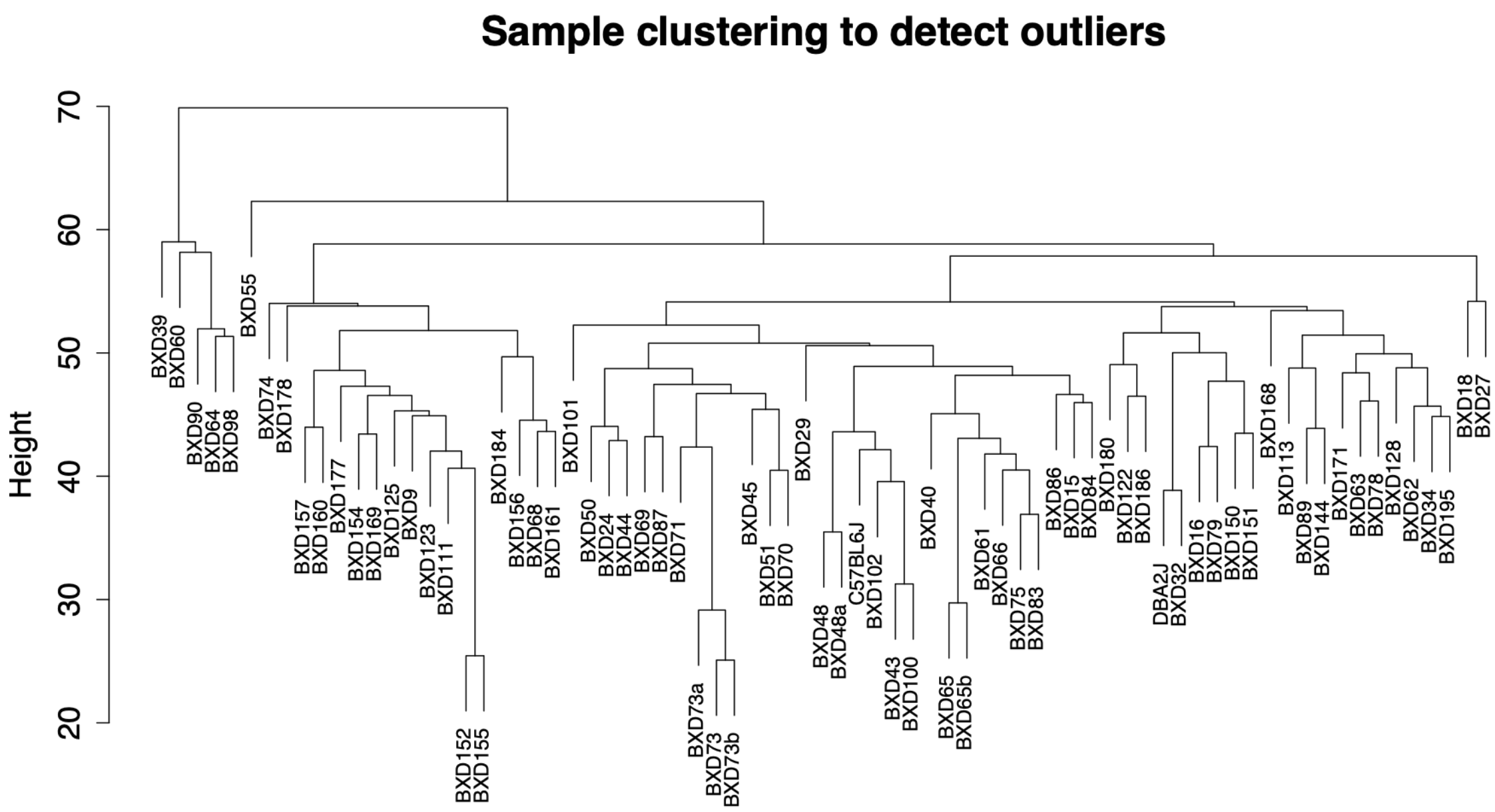

Figure S1. Clustering of BXD strains to detect outliers prior to network construction.

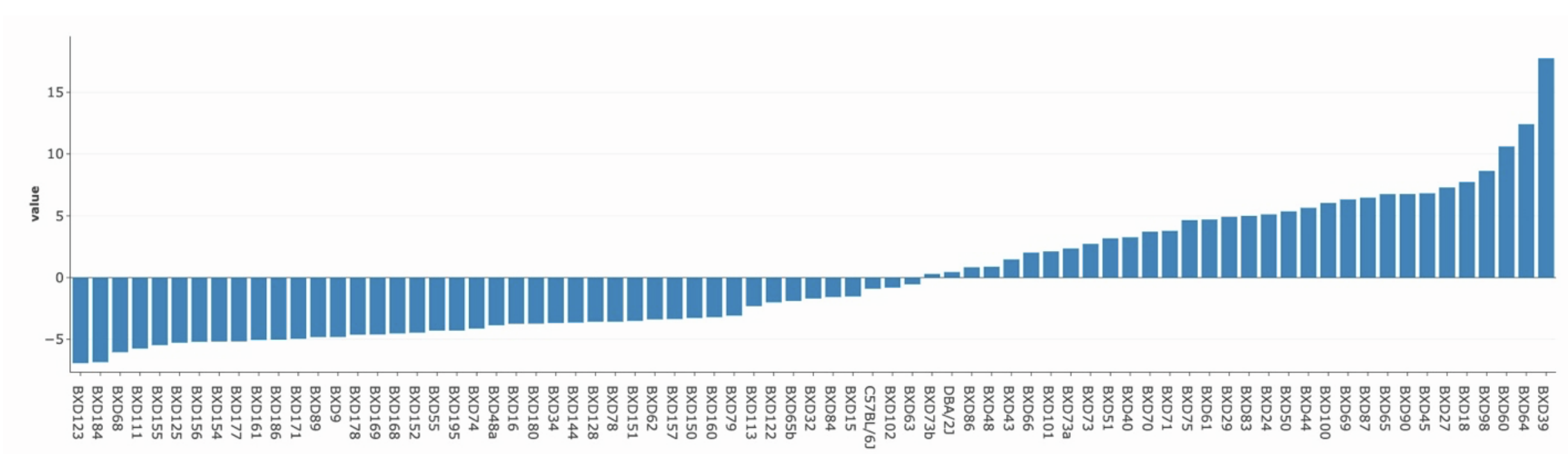

Figure S2. Distribution of iron-geneset PC1 across BXD strains. The x-axis shows BXD strains and y-axis shows the PC1 values.

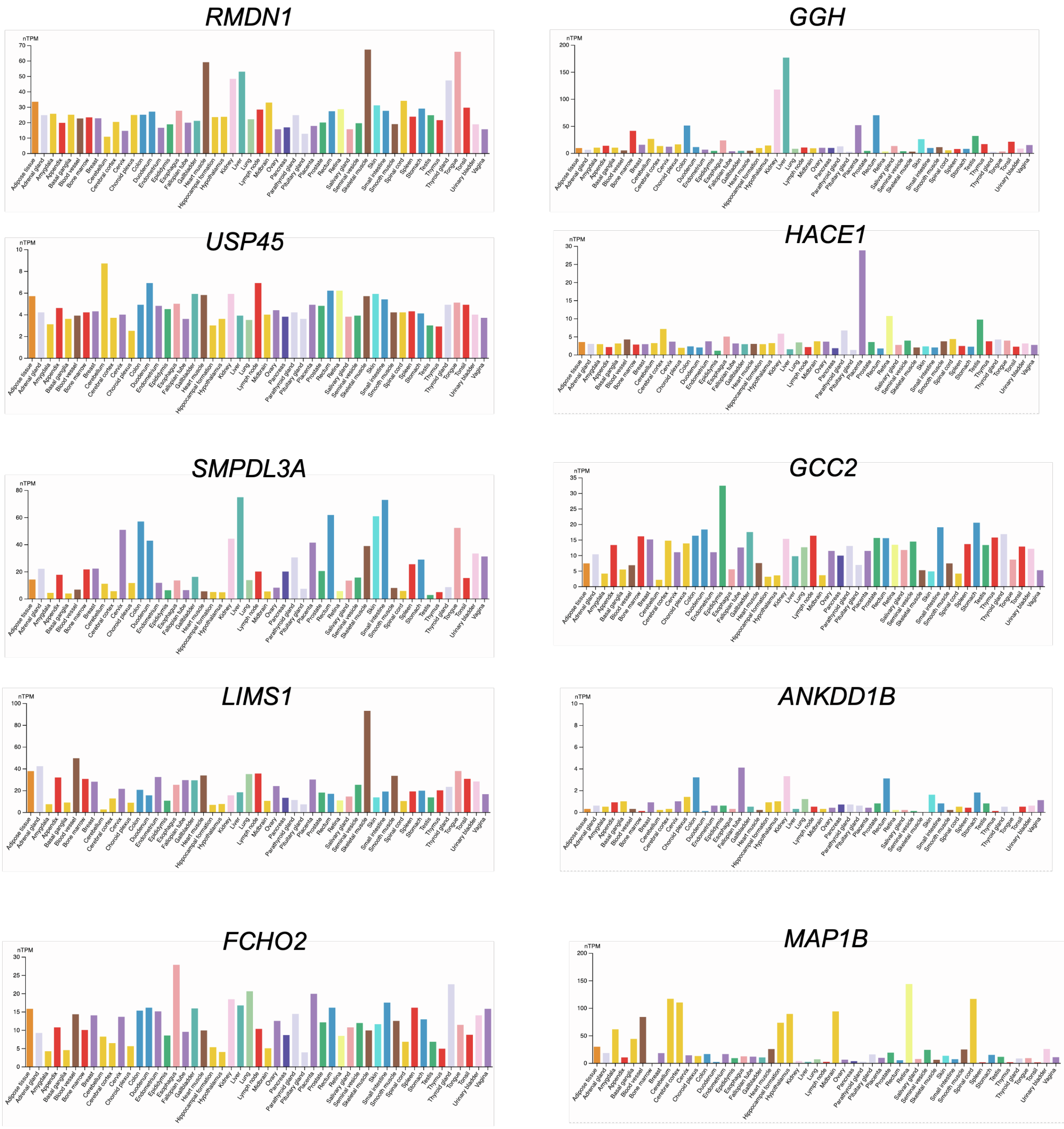

Figure S3. Human tissue expression of the selected candidate genes across three chromosomal loci. The data was collected from the Human Protein Atlas database (https://www.proteinatlas.org/).

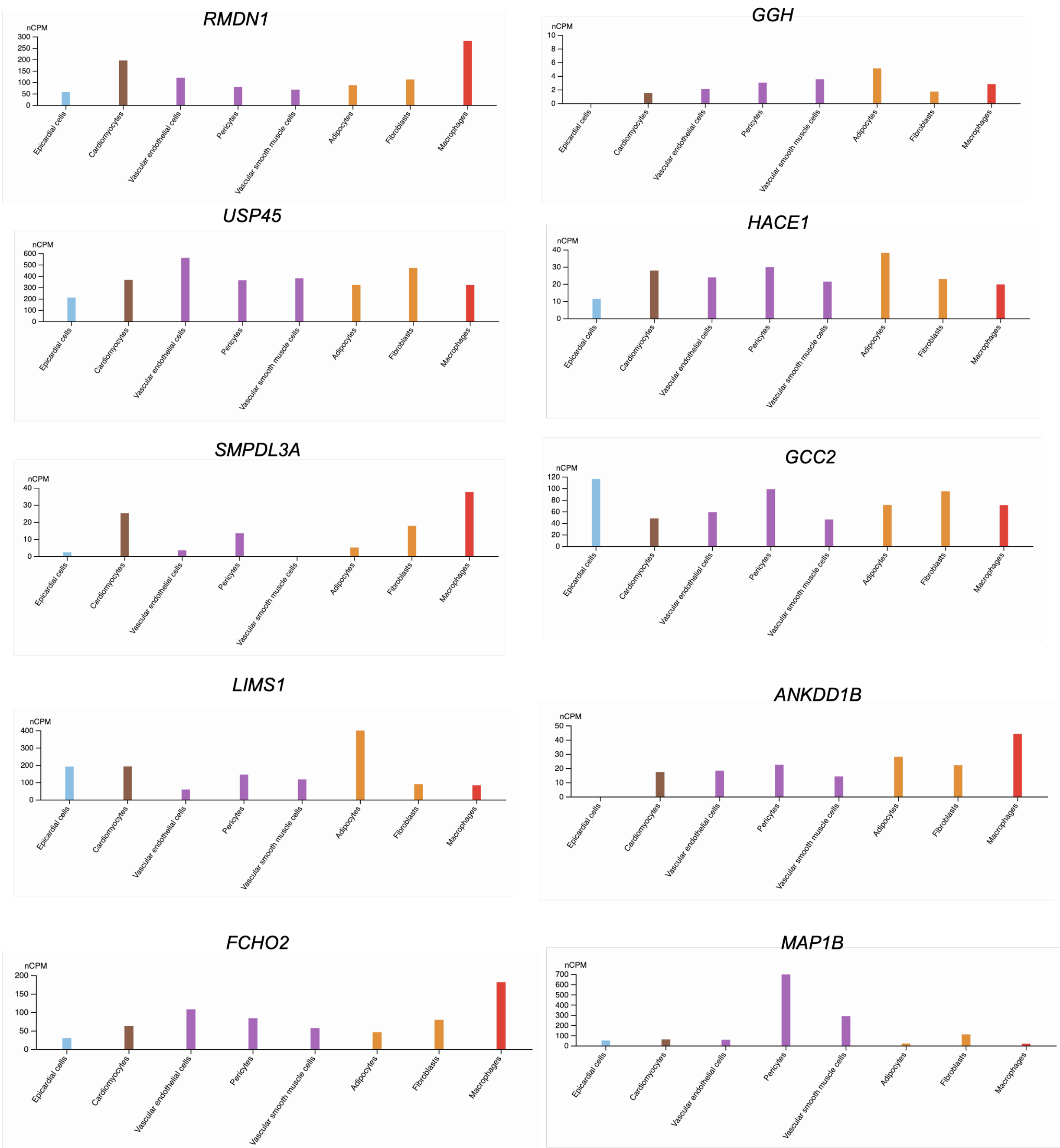

Figure S4. Human heart cell type expression of the selected candidate genes across three chromosomal loci. The data was collected from the Human Protein Atlas database (https://www.proteinatlas.org/).

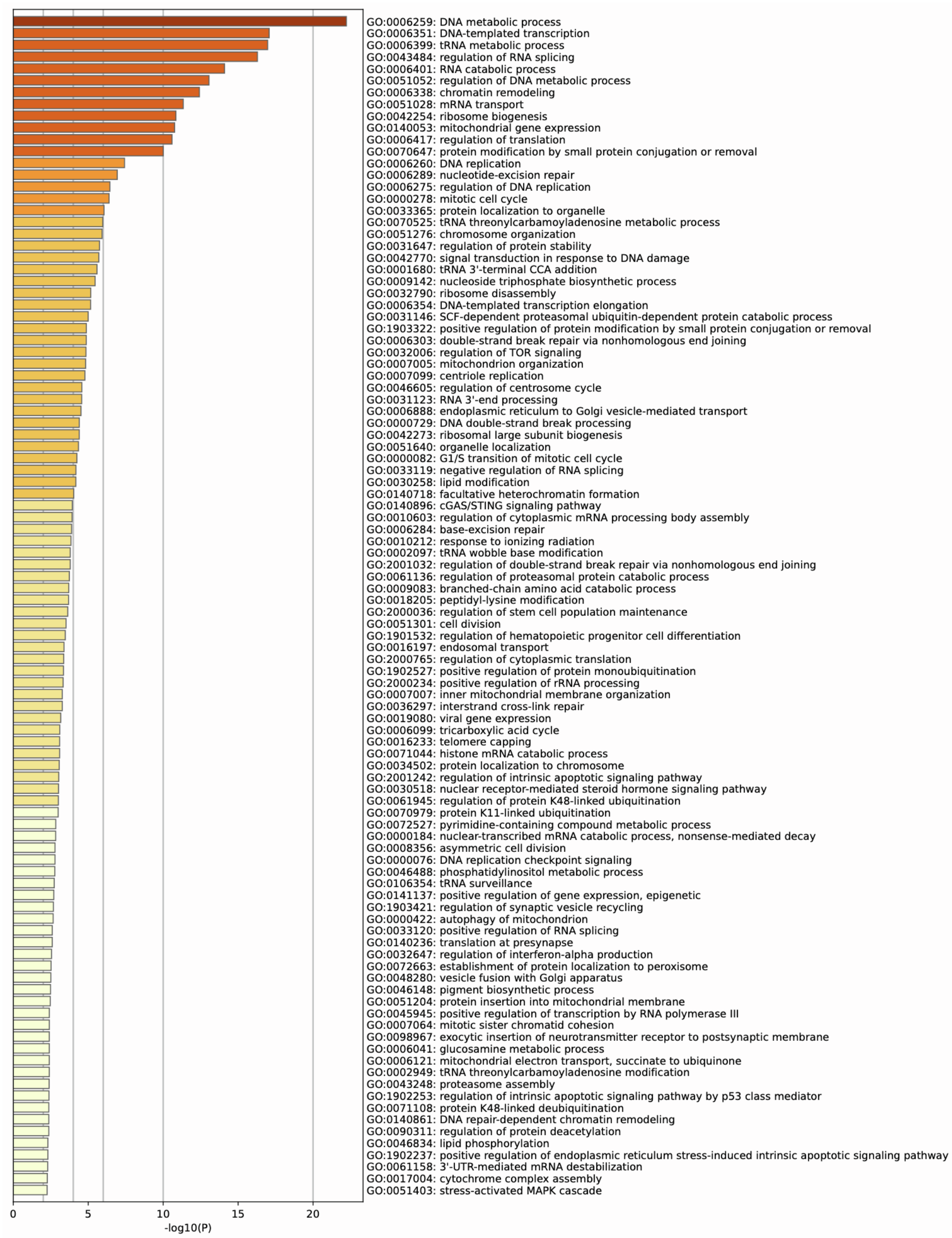

Figure S5. Top 100 Gene Ontology biological processes enriched by *Rmdn1*-correlated genes in BXD heart. The enrichment analysis was performed using Metascape (https://metascape.org/).

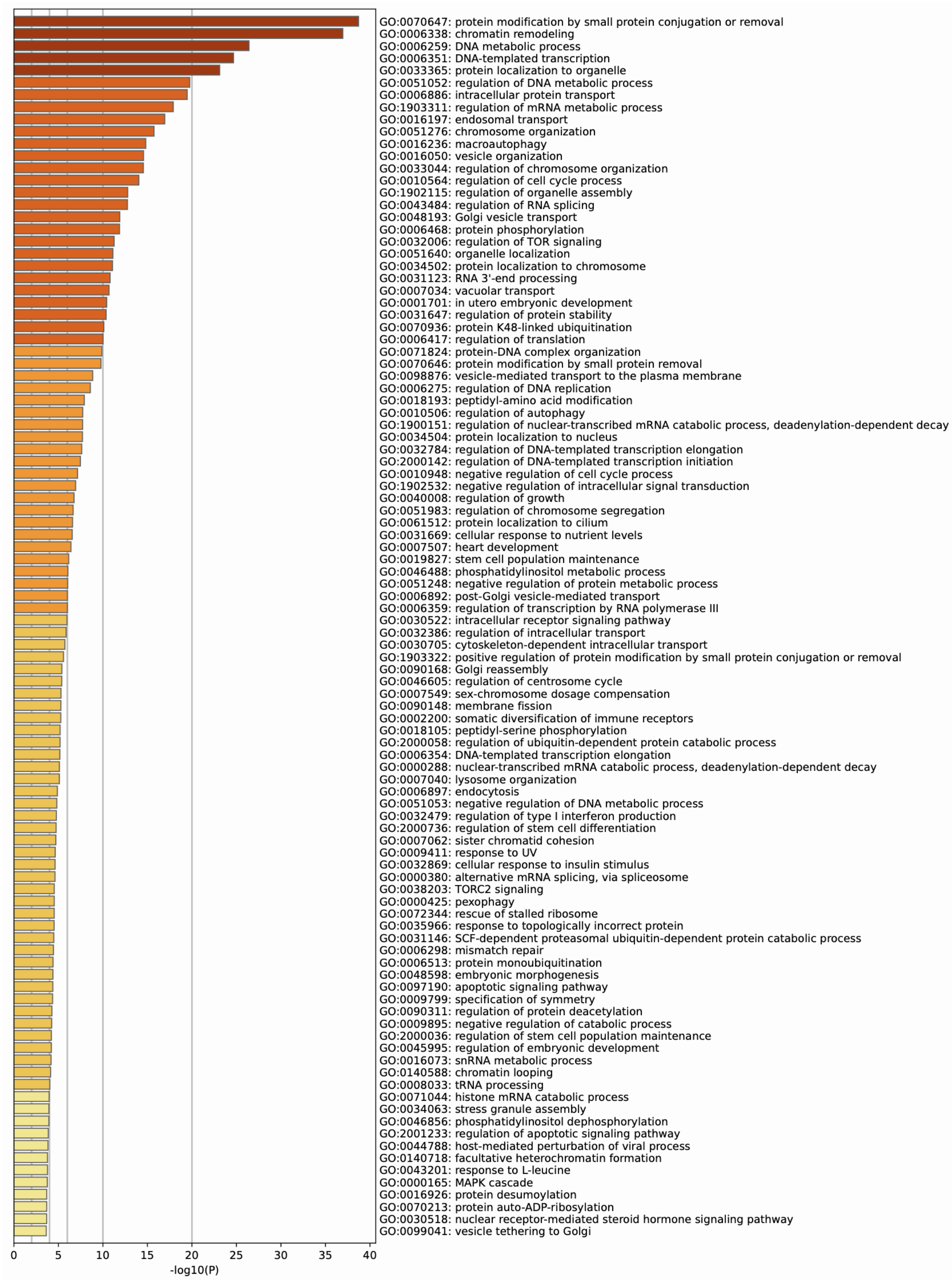

Figure S6. Top 100 Gene Ontology biological processes enriched by *Gcc2*-correlated genes in BXD heart. The enrichment analysis was performed using Metascape (https://metascape.org/).

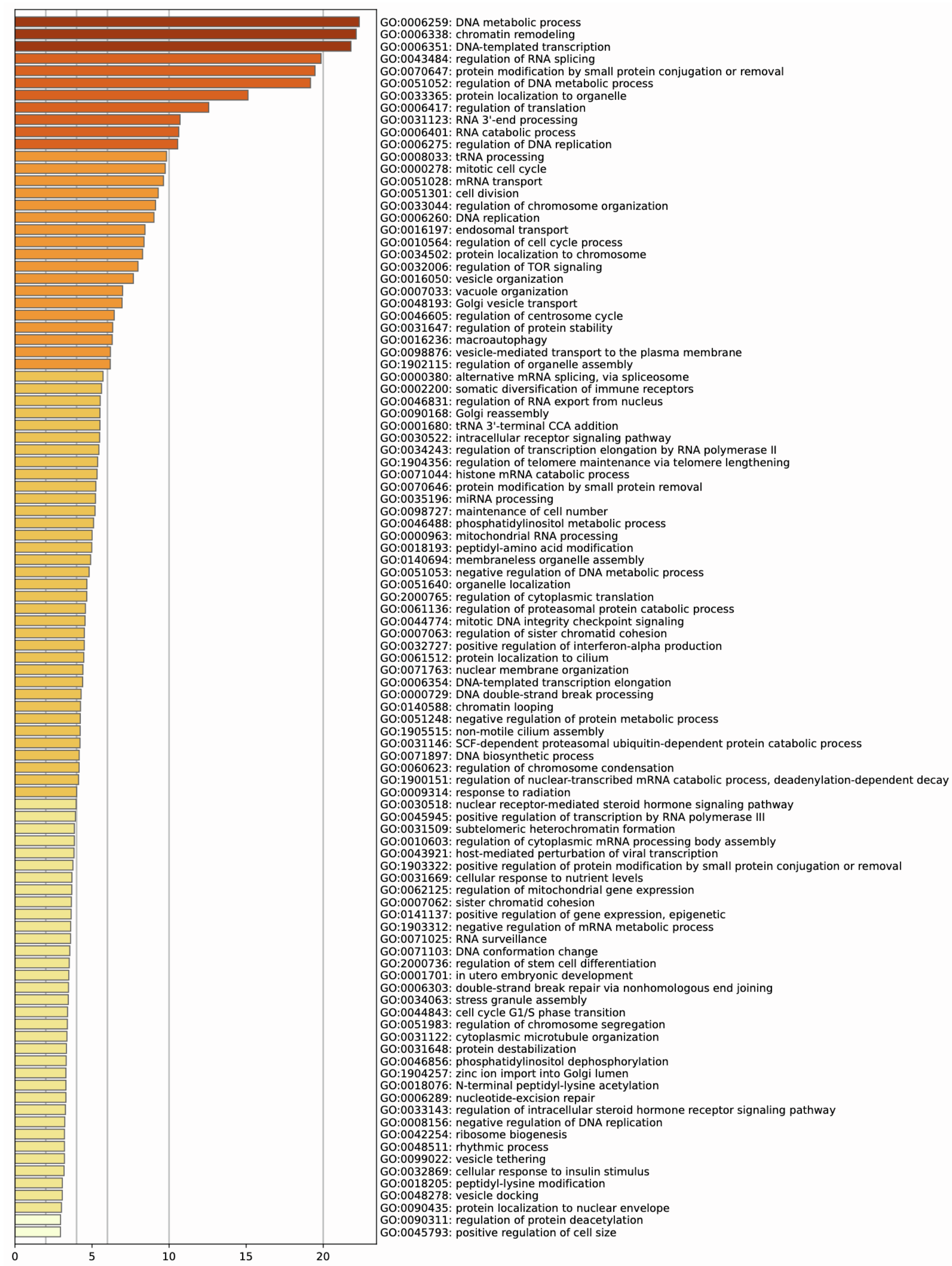

Figure S7. Top 100 Gene Ontology biological processes enriched by *Fcho2*-correlated genes in BXD heart. The enrichment analysis was performed using Metascape (https://metascape.org/).

Table S1: Details of the heart iron level phenotypes

Record ID	BXD_21394	BXD_21397	BXD_21400	BXD_21518
Description	Iron level in heart of 20 to 650-day-old males fed Envigo diet 7912 containing 240, 93, and 63 ppm Fe, Cu, and Zn, respectively (Updated) [ug/g]	Iron level in heart of 20 to 650-day-old females fed Envigo diet 7912 containing 240, 93, and 63 ppm Fe, Cu and Zn, respectively (Updated) [ug/g]	Iron concentration in heart of 20 to 240-day-old males and females fed Envigo diet 7912 containing 240, 93, and 63 ppm Fe, Cu, and Zn, respectively [ug/g]	Iron concentration in heart of 20-650-day-old males and females fed Envigo diet 7912 containing 240, 93, and 63 ppm Fe, Cu and Zn, respectively [ug/g]
Short Name	Iron heart Male Old	Iron heart Female Old	Iron heart MF Young	Iron heart MF Old
Mean Value	82.23	82.27	72.79	79.85
No. of Strains	75	80	41	83
No. of Animals	202	254	150	456
Median Age	312 days	330 days	179 days	322 days
No. of Males	202	NA	70	202
No. of Females	NA	254	80	254

Supplementary Text: Analytical Codes

```
#================================================================
# WGCNA BXD FINAL CONSOLIDATED PIPELINE (WITH MANUAL OUTLIERS)
#================================================================
library(WGCNA)
library(data.table)
library(dplyr)

options(stringsAsFactors = FALSE)
enableWGCNAThreads()

# --- HELPER FUNCTION: SAVE DUAL FORMATS ---
save_plot <- function(plot_func, filename_base, w = 10, h = 7) {
  pdf(paste0(filename_base, ".pdf"), width = w, height = h)
  plot_func()
  dev.off()
  png(paste0(filename_base, ".png"), width = w*300, height = h*300, res = 300)
  plot_func()
  dev.off()
}

# 1. LOAD DATA
raw_data <- read.csv("GN1028_Heart_BXD_Top10k_Genes.csv", row.names = 1)
datExpr <- as.data.frame(t(raw_data))

# SAVE FIGURE 01: Initial Sample Clustering (To verify outliers)
save_plot(function() {
  sampleTree = hclust(dist(datExpr), method = "average")
  plot(sampleTree, main = "Sample clustering to detect outliers",
       sub="", xlab="", cex.lab = 1.5, cex.axis = 1.5, cex.main = 2)
}, "01_Sample_Clustering_Initial", w = 13, h = 8)

# --- MANUAL OUTLIER REMOVAL ---
# This block is now back in place. Update the BXD IDs as needed.
outliers <- c("BXD39", "BXD60", "BXD64", "BXD90", "BXD98", "BXD55")
datExpr <- datExpr[!(rownames(datExpr) %in% outliers), ]

# 2. LOAD TRAIT DATA & ALIGNMENT
datTraits <- read.csv("Iron_level_BXD.csv")
rownames(datTraits) <- datTraits$Strain
targetTraits <- c("BXD_21394", "BXD_21397", "BXD_21400", "BXD_21518")

# Defensive Alignment
commonStrains <- intersect(rownames(datExpr), rownames(datTraits))
if(length(commonStrains) == 0) {
  rownames(datTraits) <- paste0("BXD", datTraits$Strain)
  commonStrains <- intersect(rownames(datExpr), rownames(datTraits))
}
datExpr <- datExpr[commonStrains, ]
```

```
datTraits_sub <- datTraits[commonStrains, targetTraits, drop = FALSE]

# 3. POWER SELECTION & DIAGNOSTICS
powers <- c(1:10, seq(12, 20, 2))
sft <- pickSoftThreshold(datExpr, powerVector = powers, networkType = "signed", verbose = 5)

# SAVE FIGURE 02: Scale Independence & Connectivity
# Adjusted for high-visibility font sizes
save_plot(function() {
  par(mfrow= c(1,2), mar = c(5,5,4,2)) # Added margin for larger axis labels

  # --- Plot 1: Scale Independence ---
  plot(sft$fitIndices[,1], -sign(sft$fitIndices[,3])*sft$fitIndices[,2],
       xlab="Soft Threshold (Power)", ylab="Scale Free Topology Model Fit, signed R^2",
       type="n", main="Scale independence",
       ylim=c(-1,1), # Adjusted ylim to 1 to see the 0.8 threshold clearly
       cex.main = 2, cex.lab = 1.7, cex.axis = 1.6)

  # Increase cex here to make the red power numbers larger
  text(sft$fitIndices[,1], -sign(sft$fitIndices[,3])*sft$fitIndices[,2],
       labels=powers, col="red", cex = 1.6)

  # Standard WGCNA threshold line is 0.8 or 0.9
  abline(h=0.80, col="red", lwd=2)

  # --- Plot 2: Mean Connectivity ---
  plot(sft$fitIndices[,1], sft$fitIndices[,5],
       xlab="Soft Threshold (Power)", ylab="Mean Connectivity",
       type="n", main="Mean connectivity",
       cex.main = 2, cex.lab = 1.7, cex.axis = 1.6)

  # Increase cex here to match
  text(sft$fitIndices[,1], sft$fitIndices[,5],
       labels=powers, col="red", cex = 1.6)

}, "02_Scale_Independence_Diagnostic", w = 10, h = 7)

# 4. NETWORK CONSTRUCTION
softPower = 12
adjacency <- adjacency(datExpr, power = softPower, type = "signed")
TOM <- TOMsimilarity(adjacency, TOMType = "signed")
dissTOM <- 1 - TOM
geneTree <- hclust(as.dist(dissTOM), method = "average")

# Module detection & Merging
dynamicMods = cutreeDynamic(dendro = geneTree, distM = dissTOM, deepSplit = 3,
                            pamRespectsDendro = FALSE, minClusterSize = 30)
dynamicColors = labels2colors(dynamicMods)
merge = mergeCloseModules(datExpr, dynamicColors, cutHeight = 0.10, verbose = 3)
moduleColors = merge$colors
MEs = orderMEs(merge$newMEs)
```

```
# --- CRITICAL: EXCLUDE GREY MODULE ---
if ("MEgrey" %in% names(MEs)) {
  MEs <- MEs[, names(MEs) != "MEgrey"]
}
isNotGrey <- moduleColors != "grey"
datExpr_filtered <- datExpr[, isNotGrey]
moduleColors_filtered <- moduleColors[isNotGrey]
nSamples <- nrow(datExpr)

# 5. MODULE-TRAIT RELATIONSHIPS & REMAINING FIGURES
moduleTraitCor <- cor(MEs, datTraits_sub, use = "p")
moduleTraitPvalue <- corPvalueStudent(moduleTraitCor, nSamples)
moduleTraitFDR <- matrix(p.adjust(moduleTraitPvalue, method = "fdr"),
                         nrow = nrow(moduleTraitPvalue), ncol = ncol(moduleTraitPvalue))
dimnames(moduleTraitFDR) <- dimnames(moduleTraitPvalue)

# SAVE FIGURE 03: Final Gene Dendrogram
# Adjusted for high-visibility labels and cleaner layout
save_plot(function() {
  plotDendroAndColors(
    geneTree,
    cbind(dynamicColors, moduleColors),
    c("Dynamic", "Merged"),
    dendroLabels = FALSE,

    # --- FONT SIZE ADJUSTMENTS ---
    main = "Gene Clustering and Module Assignment",
    cex.main = 3,           # Title font size
    cex.colorLabels = 3,  # Font size for "Dynamic" and "Merged" labels on the left
    cex.lab = 2.3,          # General label font size
    cex.axis = 2.4,        # y-axis font
    marAll = c(1, 8, 3, 1), # Increase second value to ensure "Dynamic/Merged" labels aren't cut
off

    # --- VISUAL TWEAKS ---
    addGuide = TRUE,        # Adds vertical lines to help align genes with colors
    guideHang = 0.05
  )
}, "03_Gene_Dendrogram", w = 16, h = 12) # Increased width to give the tree room to "breathe"

# SAVE FIGURE 04: Module-Trait Heatmap
save_plot(function() {
  # 1. Get the raw color names (e.g., "midnightblue")
  module_colors = gsub("ME", "", names(MEs))

  # 2. Create the "Pretty" labels (e.g., "Midnightblue")
  pretty_names = paste0(toupper(substr(module_colors, 1, 1)),
                        substr(module_colors, 2, nchar(module_colors)))

  # 3. Create the text matrix
```

```
  textMatrix = paste(signif(moduleTraitCor, 2), "\n(", signif(moduleTraitPvalue, 1), ")", sep = "")
  dim(textMatrix) = dim(moduleTraitCor)

  # 4. Increase the left margin significantly (Color bars need extra space)
  # c(bottom, left, top, right)
  par(mar = c(10, 15, 5, 2))

  labeledHeatmap(Matrix = moduleTraitCor,
                 xLabels = names(datTraits_sub),

                 # --- THE COMBO THAT FORCES COLOR BARS ---
                 yLabels = pretty_names,    # This is the text displayed
                 ySymbols = module_colors,  # This is the color used for the bar
                 yColorLabels = TRUE,       # This MUST be TRUE

                 colors = blueWhiteRed(50),
                 textMatrix = textMatrix,
                 zlim = c(-1,1),
                 main = "Module-Trait Correlation",

                 # --- FONT & SPACING ---
                 cex.main = 2.5,
                 cex.lab.x = 1.5,
                 cex.lab.y = 1.5,
                 cex.text = 1.1,
                 xLabelsAngle = 45,
                 setStdMargins = FALSE) # Critical so our par(mar) is used

}, "04_Module_Trait_Heatmap", w=9, h=10)

# SAVE FIGURE 05: Eigengene Dendrogram
save_plot(function() {
  METree = hclust(as.dist(1-cor(MEs)), method = "average")
  plot(METree, main = "Clustering of Module Eigengenes", xlab = "", sub = "")
}, "05_Eigengene_Dendrogram")

# 6. FINAL STATISTICS EXPORT (FULL 10-CSV SUITE)
write.csv(moduleTraitCor, "01_ModuleTraitCor_Rvals.csv")
write.csv(moduleTraitPvalue, "02_ModuleTraitCor_Pvals.csv")
write.csv(moduleTraitFDR, "03_ModuleTraitCor_FDR.csv")
write.csv(MEs, "04_Module_Eigengenes.csv")

# GS and MM Matrices
GS <- cor(datExpr_filtered, datTraits_sub, use = "p")
GS_p <- corPvalueStudent(GS, nSamples)
write.csv(GS, "05_GeneSignificance_Rvals.csv")
write.csv(GS_p, "06_GeneSignificance_Pvals.csv")

MM <- cor(datExpr_filtered, MEs, use = "p")
MM_p <- corPvalueStudent(MM, nSamples)
write.csv(MM, "07_ModuleMembership_Rvals.csv")
```

```
write.csv(MM_p, "08_ModuleMembership_Pvals.csv")

# Intramodular Connectivity
k <- intramodularConnectivity.fromExpr(datExpr_filtered, moduleColors_filtered,
                                       networkType = "signed", power = softPower)
write.csv(k, "09_Connectivity_Intramodular.csv")

# Master Hub Table (Consolidated Summary)
MM_vec <- sapply(1:ncol(datExpr_filtered), function(i) {
  modColor <- moduleColors_filtered[i]
  colName <- if (paste0("ME", modColor) %in% colnames(MM)) paste0("ME", modColor) else
modColor
  if (colName %in% colnames(MM)) MM[i, colName] else NA
})

hubTable <- data.frame(Gene = colnames(datExpr_filtered),
                       Module = moduleColors_filtered,
                       MM = MM_vec)
hubTable <- cbind(hubTable, GS)
write.csv(hubTable, "10_Master_HubTable_Summary.csv", row.names = FALSE)

# Gene counts per module count
genesPerModule <- as.data.frame(table(moduleColors_filtered))
write.csv(genesPerModule, "Results_GeneCount_per_Module.csv", row.names = FALSE)

#save.image("WGCNA_Final_Complete_Analysis.RData")
print("Analysis Finished: Manual Outliers Removed, Grey Excluded, 5 Plots & 10 CSVs saved.")
```

```
# ==========================================
# 1. LOAD REQUIRED PACKAGES
# ==========================================
library(arrow)
library(data.table)
library(tidyverse)
library(org.Hs.eg.db) # For translating human gene symbols

# ==========================================
# 2. SETUP YOUR TARGET GENES
# ==========================================
my_genes_raw <- readLines("QTL_genes.txt")
target_genes_human <- toupper(trimws(my_genes_raw))
cat("Loaded", length(target_genes_human), "target genes.\n\n")

# ==========================================
# 3. TRANSLATE SYMBOLS TO ENSEMBL IDs
# ==========================================
cat("Translating Gene Symbols to Ensembl IDs...\n")
```

```
gene_map <- select(org.Hs.eg.db,
                   keys = target_genes_human,
                   columns = c("ENSEMBL", "SYMBOL"),
                   keytype = "SYMBOL") %>%
  filter(!is.na(ENSEMBL))

my_ensembl_ids <- gene_map$ENSEMBL

# ============================================
# 4. LOAD ALL THREE TISSUES (Lightning Fast)
# ============================================
cat("Reading and filtering Parquet files...\n")

# Left Ventricle (Using phenotype_id)
lv_data <- read_parquet("Heart_Left_Ventricle.v11.eQTLs.signif_pairs.parquet") %>%
  mutate(gene_id_clean = str_remove(phenotype_id, "\\..*$")) %>%
  filter(gene_id_clean %in% my_ensembl_ids) %>%
  mutate(gene_name = gene_map$SYMBOL[match(gene_id_clean, gene_map$ENSEMBL)])

# Atrial Appendage
aa_data <- read_parquet("Heart_Atrial_Appendage.v11.eQTLs.signif_pairs.parquet") %>%
  mutate(gene_id_clean = str_remove(phenotype_id, "\\..*$")) %>%
  filter(gene_id_clean %in% my_ensembl_ids) %>%
  mutate(gene_name = gene_map$SYMBOL[match(gene_id_clean, gene_map$ENSEMBL)])

# Coronary Artery
ca_data <- read_parquet("Artery_Coronary.v11.eQTLs.signif_pairs.parquet") %>%
  mutate(gene_id_clean = str_remove(phenotype_id, "\\..*$")) %>%
  filter(gene_id_clean %in% my_ensembl_ids) %>%
  mutate(gene_name = gene_map$SYMBOL[match(gene_id_clean, gene_map$ENSEMBL)])

# Consolidate the exact SNPs we need to translate across all three tissues
all_needed_variants <- unique(c(lv_data$variant_id, aa_data$variant_id, ca_data$variant_id))

cat("Found eQTLs across all tissues. We only need to translate", length(all_needed_variants),
"unique SNPs!\n\n")

# ============================================
# 5. LOAD THE GTEX LOOKUP TABLE (Low Memory)
# ============================================
cat("Scanning the massive GTEx Lookup Table...\n")

lookup_table <- fread(
  "GTEx_Analysis_2021-02-11_v11_WholeGenomeSeq_953Indiv.lookup_table.txt.gz",
  select = c("variant_id", "rs_id_dbSNP155_GRCh38p13")
)

# Instantly drop the millions of rows we don't need
lookup_table <- lookup_table[variant_id %in% all_needed_variants]
cat("Dictionary loaded and minimized.\n\n")
```

```
# ==========================================
# 6. CREATE THE FORMATTING FUNCTION (FIXED)
# ==========================================
format_for_MR <- function(tissue_data, output_name) {

  merged_data <- left_join(tissue_data, lookup_table, by = "variant_id")

  formatted_data <- merged_data %>%
    separate(variant_id, into = c("chr", "pos", "ref", "alt", "build"),
             sep = "_", remove = FALSE) %>%
    dplyr::select(                        # <--- FIXED LINE HERE
      gene_name = gene_name,
      SNP = rs_id_dbSNP155_GRCh38p13,
      effect_allele = alt,
      other_allele = ref,
      beta = slope,
      se = slope_se,
      pval = pval_nominal,
      eaf = af
    ) %>%
    filter(!is.na(SNP) & SNP != ".")

  write.csv(formatted_data, paste0(output_name, ".csv"), row.names = FALSE)
  cat("[\u2713] Saved:", paste0(output_name, ".csv"), "with", nrow(formatted_data),
"independent eQTLs.\n")
}

# ==========================================
# 7. RUN THE FORMATTING PIPELINE
# ==========================================
cat("Formatting and saving final MR files...\n")
format_for_MR(lv_data, "MR_Ready_Left_Ventricle")
format_for_MR(aa_data, "MR_Ready_Atrial_Appendage")
format_for_MR(ca_data, "MR_Ready_Coronary_Artery")

cat("\nExtraction complete! Your system can rest now.\n")
```

## MENDELIAN RANDOMIZATION

```
# ==========================================
# 1. LOAD REQUIRED PACKAGES
# ==========================================
library(TwoSampleMR)
library(tidyverse)

# Plug your copied OpenGWAS token here!
Sys.setenv(OPENGWAS_JWT =
"eyJhbGciOiJSUzI1NiIsImtpZCI6ImFwaS1qd3QiLCJ0eXAiOiJKV1QifQ.eyJpc3MiOiJhcGkub3Bl
bmd3YXMuaW8iLCJhdWQiOiJhcGkub3Blbmd3YXMuaW8iLCJzdWIiOiJha2hpbC5iYWpwYWlA
Z21haWwuY29tIiwiaWF0IjoxNzc2MTcxMDk4LCJleHAiOjE3NzczODA2OTh9.QBiWBA_LLB9tvZ
```

```
TPsGJjkl4QKqgtf3Z8yI7uam4tWGsgwpqKnKHt-cK7oIs71exicld71rQSm9UlIXDT4d-
PKC4Sxb7fLDexWITIFk2hjFfq4pML4BQqGPNrSyU9hAFfgxh0XOCW7NcpijFk53L9MC6jcE6w2
aMrZ4UmHcfVf2MRvQCu3WqtB2d95FBIwT4HAzXNJ2GAx8IqUDMRIzLJT2W6BC5X0we8uAE
W2JrdwojaO6U_G6yhvXC7HfIKK_UPQfBOcd3vbXwg5LpS0TUXqTaaOdk2yu39rKBxxI0U2mft
CnykD6ESuVTWVDx3cZTacplIv4gVY76PZjM6EWLoBw")

cat("Authentication set! Ready to connect to OpenGWAS...\n")

# ==============================================
# 2. LOAD YOUR FORMATTED EXPOSURE DATA
# ==============================================
# CHANGE THIS FILENAME for each of your 3 tissue runs!
current_tissue_file <- "MR_Ready_Coronary_Artery.csv"

cat("Loading exposure data from:", current_tissue_file, "\n")
exposure_raw <- read.csv(current_tissue_file)

# Format for TwoSampleMR
exposure_data <- format_data(
  exposure_raw,
  type = "exposure",
  snp_col = "SNP",
  beta_col = "beta",
  se_col = "se",
  effect_allele_col = "effect_allele",
  other_allele_col = "other_allele",
  eaf_col = "eaf",
  pval_col = "pval",
  phenotype_col = "gene_name" # Groups the results by your iron genes
)

# ==============================================
# 3. LD CLUMPING (CRUCIAL STEP)
# ==============================================
# 1. Filter your existing 17,000 row dataset down to the strongest instruments
exposure_strict <- exposure_data %>%
  filter(pval.exposure < 1e-5)

cat("We have", nrow(exposure_strict), "highly significant SNPs left.\n")

# 2. Re-run the clumping on this strict dataset
exposure_clumped_strict <- clump_data(exposure_strict)

cat("After strict clumping, we have", nrow(exposure_clumped_strict), "perfect MR
instruments.\n")

# ==============================================
# 4. AUTO-EXTRACT MULTIPLE OUTCOMES
# ==============================================
# List all the disease and biomarker IDs you want to test
my_outcomes <- c(
```

```
  "ebi-a-GCST009541",        # Heart Failure
  "finn-b-I9_CARDIOMYOPATHY",# Cardiomyopathy
  "ebi-a-GCST006414", #Atrial Fibrillation
  "ieu-a-1052",              # Ferritin
  "ieu-a-1049",               # Serum Iron
  "ieu-a-1051", #Transferrin Saturation
  "ebi-a-GCST005913" #LVEF
)

cat("Fetching all Outcome data directly from the IEU OpenGWAS servers...\n")
outcome_data <- extract_outcome_data(
  snps = exposure_clumped_strict$SNP,
  outcomes = my_outcomes
)

# ==========================================
# 5. HARMONIZE THE DATA
# ==========================================
# Aligns the effect alleles between your GTEx data and the Heart Failure GWAS
cat("Harmonizing Exposure and Outcome alleles...\n")
harmonized_data <- harmonise_data(
  exposure_dat = exposure_clumped_strict,
  outcome_dat = outcome_data
)

# ==========================================
# 6. RUN THE MENDELIAN RANDOMIZATION
# ==========================================
cat("Calculating Causal Estimates for all traits...\n")
mr_results <- mr(harmonized_data)

# Generate Odds Ratios (This automatically handles both Beta and OR calculations)
mr_results_final <- generate_odds_ratios(mr_results)

output_filename <- paste0("MR_Results_", gsub(".csv", "", current_tissue_file),
"_All_Outcomes.csv")
write.csv(mr_results_final, output_filename, row.names = FALSE)
cat("[\u2713] Primary Results saved to:", output_filename, "\n")

# ==========================================
# 7. RUN SENSITIVITY ANALYSES
# ==========================================
# (These only calculate for genes that have 3 or more independent SNPs)
cat("Running Pleiotropy and Heterogeneity checks...\n")

pleiotropy <- mr_pleiotropy_test(harmonized_data)
write.csv(pleiotropy, paste0("Pleiotropy_", gsub(".csv", "", current_tissue_file), ".csv"),
row.names = FALSE)

heterogeneity <- mr_heterogeneity(harmonized_data)
```

```
write.csv(heterogeneity, paste0("Heterogeneity_", gsub(".csv", "", current_tissue_file), ".csv"),
row.names = FALSE)
```
